# Development, validation, and application of a machine learning model to estimate salt consumption in 54 countries

**DOI:** 10.7554/eLife.72930

**Published:** 2022-01-25

**Authors:** Wilmer Cristobal Guzman-Vilca, Manuel Castillo-Cara, Rodrigo M Carrillo-Larco

**Affiliations:** 1 School of Medicine Alberto Hurtado, Universidad Peruana Cayetano Heredia Lima Peru; 2 CRONICAS Centre of Excellence in Chronic Diseases, Universidad Peruana Cayetano Heredia Lima Peru; 3 Sociedad Científica de Estudiantes de Medicina Cayetano Heredia (SOCEMCH), Universidad Peruana Cayetano Heredia Lima Peru; 4 Universidad de Lima Lima Peru; 5 Department of Epidemiology and Biostatistics, School of Public Health, Imperial College London London United Kingdom; University of Padua Padua Italy; University of Zurich Zurich Switzerland

**Keywords:** artificial intelligence, deep learning, cardio-metabolic risk factors, cardiovascular health, global health, population health, Human

## Abstract

Global targets to reduce salt intake have been proposed, but their monitoring is challenged by the lack of population-based data on salt consumption. We developed a machine learning (ML) model to predict salt consumption at the population level based on simple predictors and applied this model to national surveys in 54 countries. We used 21 surveys with spot urine samples for the ML model derivation and validation; we developed a supervised ML regression model based on sex, age, weight, height, and systolic and diastolic blood pressure. We applied the ML model to 54 new surveys to quantify the mean salt consumption in the population. The pooled dataset in which we developed the ML model included 49,776 people. Overall, there were no substantial differences between the observed and ML-predicted mean salt intake (p<0.001). The pooled dataset where we applied the ML model included 166,677 people; the predicted mean salt consumption ranged from 6.8 g/day (95% CI: 6.8–6.8 g/day) in Eritrea to 10.0 g/day (95% CI: 9.9–10.0 g/day) in American Samoa. The countries with the highest predicted mean salt intake were in the Western Pacific. The lowest predicted intake was found in Africa. The country-specific predicted mean salt intake was within reasonable difference from the best available evidence. An ML model based on readily available predictors estimated daily salt consumption with good accuracy. This model could be used to predict mean salt consumption in the general population where urine samples are not available.

## Introduction

The association between high sodium/salt intake and high blood pressure, a major risk factor of cardiovascular diseases (CVDs), is well-established (He et al., 2013; World Health Organization, 2021a; Poggio et al., 2015). More than 1.7 million CVD deaths were attributed to a diet high in sodium in 2019, with ~90% of these deaths occurring in low- and middle-income countries (LMICs) (GBD 2019 Risk Factors Collaborators, 2020; GBD Results Tool, 2021). Consequently, salt reduction has been included in international goals: the World Health Organization (WHO) recommendation of limiting salt consumption to <5 g/day (World Health Organization, 2021a), and the agreement by the WHO state members of a 30% relative reduction in mean population salt intake by 2025 (WHO. World Health Organization, 2021). Because available evidence suggests that sodium/salt consumption is higher than the global targets (Powles et al., 2013; Carrillo-Larco and Bernabe-Ortiz, 2020; Oyebode et al., 2016) we need timely and consistent data of sodium/salt consumption in the general population to track progress of salt reduction targets.

Global efforts have been made to produce comparable estimates of sodium/salt intake for all countries (Powles et al., 2013). Similarly, researchers have summarized all the available evidence in specific world regions (Carrillo-Larco and Bernabe-Ortiz, 2020, Oyebode et al., 2016). Although the global endeavor was based on the gold standard method to assess sodium/salt intake (i.e., 24 hr urine sample), their estimates were up to 2010 (Powles et al., 2013). Therefore, robust and comparable sodium/salt intake estimates for all countries lack for the last 10 years. The regional endeavors summarized population-based evidence, yet they conducted study-level meta-analyses in which the original studies could have followed different laboratory methods, and they did not study all countries in the region. Therefore, comparability across studies could be limited and evidence lacks for many countries. Finding a method to estimate sodium/salt consumption in national samples leveraging on available data is needed to update and complement the existing evidence (Powles et al., 2013; Carrillo-Larco and Bernabe-Ortiz, 2020; Oyebode et al., 2016; Thout et al., 2019). Quantifying sodium/salt intake based on 24 hr urine samples is costly and burdensome, limiting its use in population-based studies or national health surveys. As an alternative, equations have been developed to estimate sodium/salt intake based on spot urine (SU) samples (Brown et al., 2013; Kawasaki et al., 1993; Toft et al., 2014; Tanaka et al., 2002). Although these equations may not deliver identical results to those based on 24 hr urine samples at the individual level, at the population level the difference between SU samples and 24 hr samples appears to be small (Huang et al., 2016; Santos et al., 2020). However, these equations have been used in few WHO STEPS and other national health surveys (World Health Organization, 2021b), leaving several countries without data to quantify the local sodium/salt consumption because they do not have access to SU samples (World Health Organization, 2021c).

If we could (accurately) estimate sodium/salt intake at the population level based on variables that are routinely available in national health surveys (e.g., weight or blood pressure), mean sodium/salt intake at the population level in countries that currently lack urine data (i.e., 24 hr or spot) could be computed using these available predictors. Advanced analytic techniques like machine learning (ML) could make accurate predictions and inform about the mean sodium/salt intake at the population level. We developed an ML predictive model to estimate mean salt intake at the population level (not at the individual level) using routinely available variables in national health surveys. We applied this ML model to other national health surveys without urine data to compute the mean salt intake in the general population.

## Results

### Study population for model derivation and validation

The pooled dataset included 49,776 people from 21 surveys in 19 countries (i.e., two countries, Bhutan and Mongolia, had two surveys) conducted between 2013 and 2019 (Appendix 1—table 1). Overall, the mean age ranged from 33 (95% confidence interval [95% CI]: 33–34) years in Zambia to 43 (95% CI: 42–44) years in Belarus. The proportion of men ranged from 35.7% in Tonga to 61.4% in Solomon Islands. The mean SBP was lowest in Jordan (117.7 mmHg [95% CI: 115.7–119.8 mmHg]) and highest in Belarus (134.6 mmHg [95% CI: 133.6–135.5 mmHg]). The mean DBP was lowest in Chile (73.6 mmHg [95% CI: 72.5–74.6 mmHg]) and highest in Belarus (84.9 mmHg [95% CI: 84.4–85.5 mmHg]). The mean weight ranged from 54.6 kg (95% CI: 53.8–55.5 kg) in Nepal to 98.6 kg (95% CI: 97.7–99.5 kg) in Tonga. The mean height ranged from 1.55 m (95% CI: 1.55–1.56 m) in Nepal to 1.71 m (95% CI: 1.70–1.71 m) in Tokelau.

### Observed and predicted mean salt intake during the ML model derivation and validation

**Figure 1.**
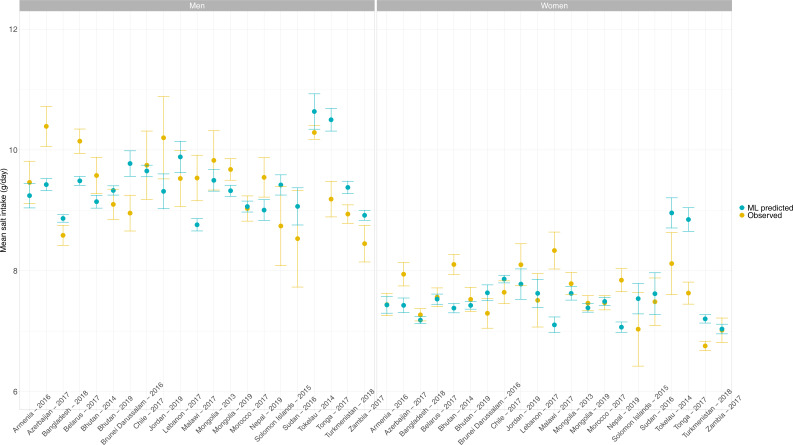
Observed and predicted mean salt intake (g/day) by sex in each survey included in the machine learning (ML) model development. Exact estimates (along with their 95% CI) are presented in Appendix 1—table 2. These results were computed with the test dataset only. Results are for the HuR algorithm, which was the model with the best performance.

In the test dataset including 20 WHO STEPS surveys and one national health survey (Chile) (i.e., 21 surveys in total), the observed mean salt intake computed as per the INTERSALT equation was higher in men than in women in all countries; it ranged from 8.5 g/day (95% CI: 8.2–8.8 g/day; Zambia) to 10.4 g/day (95% CI: 10.1–10.7 g/day; Azerbaijan) in men and from 6.8 g/day (95% CI: 6.7–6.8 g/day; Turkmenistan) to 8.3 g/day (95% CI: 8.0–8.6 g/day; Malawi) in women. Across countries, the predicted mean salt intake was also higher in men than in women. Results for each survey are presented in Figure 1 and Appendix 1—table 2.

The mean observed salt intake was higher in people aged ≥30 years (7.9 g/day vs. 8.4 g/day, p<0.05 for independent *t*-test), and so was for people with raised blood pressure (≥140/90 mmHg) (8.7 g/day vs. 8.2 g/day, p<0.05). The mean salt consumption was also different across body mass index (BMI) categories (p<0.05 for ANOVA test). The same profile was found for predicted mean salt intake (Appendix 1—table 3).

In men across all countries in the test dataset including 20 WHO STEPS surveys (representing 18 countries) and 1 national health survey (Chile), the mean difference between observed and predicted mean salt intake was –0.02 g/day (p<0.001 for paired *t*-test). Across all surveys, the positive mean difference farthest from zero was 0.54 g/day (Nepal, p<0.001 for paired *t*-test), and the negative mean difference farthest from zero was –1.31 g/day (Tonga, p<0.001 for paired *t*-test). The mean difference closest to zero was –0.03 g/day (Morocco, p=0.308 for paired *t*-test) (Appendix 1—table 4).

In women across all countries in the test dataset including 20 WHO STEPS surveys (representing 18 countries) and 1 national health survey (Chile), the mean difference between the observed and predicted mean salt intake was 0.01 g/day (p<0.001 for paired *t*-test). The positive mean difference farthest from zero was 1.23 g/day (Malawi, p<0.001 for paired *t*-test) and the negative mean difference farthest from zero was in –1.22 g/day (Tonga, p<0.001 for paired *t*-test). The mean difference closest to zero was 0.01 g/day (Armenia, p=0.195 for paired *t*-test) (Appendix 1—table 4).

None of the countries herein analyzed, regardless of the method of sodium intake assessment (i.e., observed or predicted), showed a mean salt intake below the WHO recommended level of <5 g/day (Figure 1, Appendix 1—table 2). The same occurred for the mean salt intake estimates using the Kawasaki, Toft, and Tanaka formulas (Appendix 1—table 5).

### Implementation of the developed ML model to predict salt consumption in 54 countries

The pooled dataset where we applied the ML model included 166,677 people from 54 countries in 54 WHO STEPS surveys conducted between 2004 and 2018 (Appendix 1—table 6). Overall, the mean age ranged from 31 (95% CI: 31–32) years in Ethiopia to 43 (95% CI: 40–47) years in Barbados. The proportion of men ranged from 17.2% in Eritrea to 63.8% in Timor-Leste. The mean SBP was lowest in Cambodia (116.2 mmHg [95% CI: 115.6–116.9 mmHg]) and highest in Mozambique (138.7 mmHg [95% CI: 136.3–141.0 mmHg]). The mean DBP was lowest in Cambodia (72.4 mmHg [95% CI: 71.8–73.0 mmHg]) and highest in Kyrgyzstan (86.8 mmHg [95% CI: 85.9–87.8 mmHg]). The mean weight ranged from 51.8 kg (95% CI: 51.2–52.4 kg) in Eritrea to 100.4 kg (95% CI: 100.1–100.8 kg) in American Samoa. The mean height ranged from 1.54 m (95% CI: 1.54–1.55 m) in Lao People’s Democratic Republic to 1.70 m (95% CI: 1.70–1.71 m) in British Virgin Islands.

**Figure 2.**
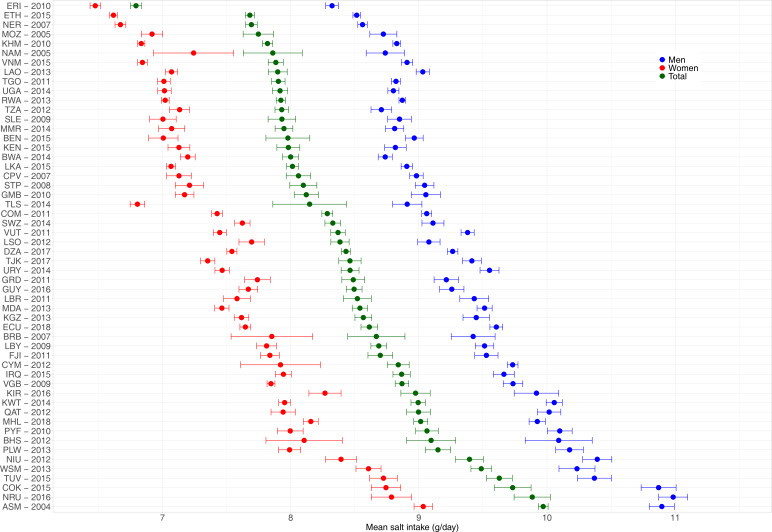
Predicted mean salt intake (g/day) by sex in each of the 54 national surveys included in the application of the model herein developed. Exact estimates (along with their 95% CI) are presented in Appendix 1—table 7. Countries are presented in ascending order based on their overall mean salt intake (i.e., countries with the highest mean salt intake are at the bottom).

Across the 54 countries, the overall predicted mean salt intake ranged from 6.8 g/day (95% CI: 6.8–6.8 g/day) in Eritrea to 10.0 g/day (95% CI: 9.9–10.0 g/day) in American Samoa. The mean was always higher in men than in women. None of the countries herein analyzed, regardless of sex, showed a predicted mean salt intake below the WHO recommended level of <5 g/day (Figure 2, Appendix 1—table 7).

In men, the countries with the highest predicted mean salt intakes were Nauru (11.0 g/day), American Samoa and Cook Islands (both with 10.9 g/day), and Niue and Tuvalu (both with 10.4 g/day); remarkably, all of these countries are in the Western Pacific. In contrast, the lowest predicted mean salt intake in men was in Eritrea (8.3 g/day), Ethiopia (8.5 g/day), and Niger (8.6 g/day); remarkably, all of these countries are in Africa.

In women, the countries with the highest predicted mean salt intake were American Samoa (9.0 g/day), Nauru (8.8 g/day), and Cook Islands and Tuvalu (both with 8.7 g/day); all of these countries are in the Western Pacific. Conversely, the lowest predicted mean salt intake in women was in Eritrea (6.5 g/day), Ethiopia (6.6 g/day), and Niger (6.7 g/day); all of these countries are in Africa.

## Discussion

### Main findings

This work leveraged on 21 national health surveys and readily available predictors to develop an ML model to predict salt consumption; this model was then applied to national surveys in 54 countries. It should be noted that we analyzed SU samples. These are not the gold standard to assess salt consumption. Results should be interpreted in light of this limitation, considering that our model aimed to deliver estimates at the population level (not individual level) (Huang et al., 2016; Santos et al., 2020). The HuR ML algorithm yielded the predictions closest to the observed salt intake: the mean difference between predicted and observed salt consumption across surveys was –0.02 g/day in men and 0.01 g/day in women. We used this novel ML model to predict salt consumption in 54 countries, where the mean salt consumption ranged from 8.3 g/day (Eritrea) to 11.0 g/day (Nauru) in men; these numbers in women ranged from 6.5 g/day (Eritrea) to 9.0 g/day (American Samoa). This work aimed to elaborate on novel analytical tools to predict salt consumption where national surveys have not collected this information, limiting their ability to keep track of mean sodium consumption in the general population. Pending external independent validation, our model could be used in monitoring frameworks of salt consumption because most countries do not collect sodium samples in their national health surveys. Our model could contribute to the global surveillance of salt consumption, a relevant cardiometabolic risk factor (He et al., 2013; World Health Organization, 2021a; Poggio et al., 2015).

### Public health implications

ML models have been used extensively to predict relevant clinical outcomes (e.g., mortality) and epidemiological indicators (e.g., forecasting COVID-19 cases) (Wang et al., 2020; Wynants et al., 2020; Groot et al., 2021; Watson et al., 2021; Mohan et al., 2021). Furthermore, ML algorithms have proven to be useful for understanding complex outcomes (e.g., identifying clusters of people with diabetes) based on simple predictors (e.g., BMI) in nationally representative survey data (Oh et al., 2019; García de la Garza et al., 2021; Carrillo-Larco et al., 2021). Our work complements the current evidence on ML algorithms by demonstrating its use in a relevant field: population salt consumption. In so doing, we delivered a pragmatic tool that could be used to inform the surveillance of salt consumption in countries where national surveys do not objectively collect this information (e.g., SU samples). Moreover, this work provided preliminary evidence to update the global estimates of population-based sodium consumption (Powles et al., 2013) by informing about the mean sodium consumption in 54 countries. Our results suggest that mean salt consumption is above the WHO recommended level in all the 54 countries herein analyzed, and it was the highest among countries in the Western Pacific, and the lowest among countries in Africa. This finding, which is consistent with a global work (Powles et al., 2013), calls for urgent actions to reduce salt consumption in these 54 countries, especially those in the Western Pacific.

We do not believe that our – or any other – ML model should replace a comprehensive population-based nationally representative health survey with 24 hr or SU samples. However, until such surveys are available in many countries and periodically conducted, we could suggest using an estimation approach to shed lights about the mean salt consumption in the population. Our ML model seems to be a reasonably good alternative and could become a pragmatic tool for surveillance systems that keep track of sodium consumption in accordance with global goals (World Health Organization, 2021a; WHO. World Health Organization, 2021).

### Research in context

A global effort provided mean sodium/salt consumption estimates for 187 countries in 1990 and 2010 (Powles et al., 2013); they used 24 hr urine samples and dietary reports from surveys conducted in 66 countries. Unfortunately, their results were until 2010. Our results advanced this evidence by providing more recent salt consumption estimates because most of the surveys in which we applied our ML model were conducted after 2010 (Appendix 1—table 6).

Compared to the global estimates for the same countries in 2010, (Powles et al., 2013), our mean salt consumption estimates were very similar. For example, our 2010 mean salt consumption estimates for Cambodia, Eritrea, and the Gambia were 7.8 g/day, 6.8 g/day, and 8.1 g/day, whereas the estimates by Powles et al., 2013 were 11.0 g/day, 5.9 g/day, and 7.7 g/day (Appendix 1—table 8; Powles et al., 2013). We further compared our estimates for surveys conducted between 2007 and 2013 (±3 years around 2010) with the 2010 estimates provided by Powles et al., 2013, and our results were also within reasonable difference. The largest differences were in Tajikistan (8.5 by our ML model vs. 13.5 by Powles et al., 2013), as well as in Kyrgyzstan (8.6 vs. 13.4 by Powles et al., 2013) and Samoa (9.5 vs. 5.2 by Powles et al., 2013). It appears that our predictions were higher than those provided by Powles et al., 2013 in countries with presumably low salt consumption (e.g., Samoa); conversely, in countries with presumably high salt consumption (e.g., Kyrgyzstan), our predictions revealed smaller estimates than those by Powles et al., 2013 (Appendix 1—table 8). These differences could be explained by the fact that our ML model was developed based on SU samples rather than 24 hr urine samples as Powles et al., 2013 did. Strong evidence indicates that estimates based on SU may overestimate salt intake at lower levels of consumption and underestimate salt intake at higher levels of consumption (Huang et al., 2016).

In addition to the global work by Powles et al., 2013, there are other reports from some specific countries. For example, a survey conducted between 2012 and 2016 with 24 hr urine samples in Fiji and Samoa showed that the mean salt consumption was 10.6 g/day and 7.1 g/day, respectively (Santos et al., 2019). The estimates from our ML model for Fiji (2011) and Samoa (2013) suggested that the mean salt consumption was 8.7 g/day and 9.5 g/day, respectively. A survey in Vanuatu in 2016 based on 24 hr urine sample informed that the mean salt intake was 5.9 g/day (Paterson et al., 2019); our estimate for the year 2011 was 8.4 g/day. In 2009 in Vietnam, a survey with SU samples revealed that the mean salt consumption was 9.9 g/day (Jensen et al., 2018); our prediction for the year 2015 was 7.9. These comparisons suggest that our ML-predicted estimates are plausible and close to the best available evidence.

Although these comparisons do not validate our predictions in the 54 national surveys, they suggest that our salt consumption estimates are within reasonable distance from the best available evidence. Until better data are available (e.g., national survey with spot or 24 hr urine sample), our model could provide preliminary evidence to inform the national mean salt consumption. Careful interpretation is warranted to understand the strengths and limitations of our ML-based predictions.

### Strengths and limitations

We followed sound and transparent methods to develop an ML model to predict salt consumption at the individual level. We leveraged on open-access national data collected following standard and consistent protocols (World Health Organization, 2021b; Departamento de Epidemiologia. Ministerio de Salud, 2021). Most of the surveys we analyzed were conducted after 2010, providing more recent evidence than the latest global effort to quantify salt consumption in all countries (Powles et al., 2013). Notwithstanding, we must acknowledge some limitations. First and foremost, urine data was based on a spot sample, which is not the gold standard (24 hr urine sample) to measure daily salt consumption. Future work should verify and advance our results using on 24 hr urine samples available in nationally representative samples; in the meantime, our work has led the foundations and hopefully sparked interest to use available data and novel analytical techniques to deliver estimates of salt consumption in the general population. While SU samples may not be the best approach to estimate salt consumption at the individual level, at the population level the means estimated based on SU samples and 24 hr urine samples are similar (Huang et al., 2016; Santos et al., 2020). Therefore, the limitation of using SU samples only may have had little impact on our mean estimates, which are the country level, not at the individual level. While this – reanalysis of SU sample rather than 24 hr urine samples – is a limitation of our work, it is also an observation showing the lack of nationally representative surveys with 24 hr urine samples available for independent reanalyses. Second, even though we analyzed 21 national surveys (representing 19 countries) to develop our ML model, the sample size could still be limited for a data-driven ML algorithm (i.e., 24,889 observations were included in model development). A larger and global work in which all relevant data sources are pooled is needed; while this endeavor takes place, our work has provided recent estimates of salt consumption at the population level in 54 countries. In this line, there are still countries that were not herein included. Researchers in these countries, along with local (e.g., ministries of health) and international health authorities (e.g., WHO), should conduct studies/surveys to collect data on salt consumption. This would inform global targets but also local needs and interventions.

An ML model based on readily available variables was accurate to predict daily salt consumption. This ML model applied to 54 national surveys with no urine samples to compute daily salt consumption revealed high levels of salt intake particularly in the Western Pacific region. Pending further validation, this ML model could be used to keep track of the overall sodium consumption where resources are not available to conduct national surveys with urine samples.

## Methods

### Study design

This is an individual-level data pooling ML analysis.

### Data sources

We sought surveys that met these two criteria: (i) nationally representative health surveys (i.e., community or subnational surveys were not included); and (ii) surveys that were open access or that could be accessed without significant administrative burden (e.g., data sharing agreements that may involve institutional signatures).

First, we downloaded 20 WHO STEPS surveys and 1 national health survey with SU samples; these surveys were used for the training, validation, and testing of the ML model. These 21 surveys represented 19 countries; two countries contributed with two surveys: Bhutan 2014 and 2019 as well as Mongolia 2013 and 2019. Second, we downloaded 54 new WHO STEPS surveys that had the variables included in the ML prediction model (see ‘Variables’ section), but did not have SU samples. The ML model herein developed was applied to these 54 surveys to estimate the mean salt consumption in the population.

To identify additional data sources, we searched the original publications included in one global analysis (Powles et al., 2013) and three systematic reviews about sodium/salt consumption at the population level (Carrillo-Larco and Bernabe-Ortiz, 2020; Oyebode et al., 2016; Thout et al., 2019). This search led to the identification of the national health survey included in the model derivation. All other data sources included in those references (Powles et al., 2013; Carrillo-Larco and Bernabe-Ortiz, 2020; Oyebode et al., 2016; Thout et al., 2019) did not meet our selection criteria.

In conclusion, our ML model was developed based on 21 surveys (20 WHO STEPS and 1 national health survey). Then, our ML model was applied to 54 WHO STEPS survey to compute the mean daily salt consumption at the population level.

According to the World Bank classification (Appendix 1—table 9), there were 9 high-income countries (2 in model derivation and 7 in model application), 16 low-income countries (1 in model derivation and 15 in model application), 26 lower-middle-income countries (9 in model derivation and 17 in model application), and 18 upper-middle-income countries (6 in model derivation and 12 model application). There were four countries (one in model derivation and three in model application) without income classification (British Virgin Islands, Cook Islands, Niue, and Tokelau).

### Rationale

We hypothesized that an ML model could accurately predict salt consumption at the individual level, to then inform the overall mean in the underlying population. In addition, we endeavored to develop an ML model with simple predictors; that is, variables that are routinely available in national health surveys contrary to urine sample that are seldom collected in national health surveys. If the model were indeed accurate, then it could be applied to national surveys without urine samples but with the relevant predictors to inform about the mean salt consumption in the overall population. These model-driven estimates could be preliminary until a national health survey is conducted to study mean salt consumption with urine samples. Ideally, salt consumption should be informed by 24 hr urine samples, which are seldom available in large population-based and nationally representative health surveys. The fact that we analyzed SU samples is a limitation of our work, and the results should be interpreted accordingly. However, we aimed to develop an ML model that can be used to predict mean estimates at the population level, not at the individual level. In other words, our model should not be applied to a patient to estimate his/her salt consumption. We did not develop a diagnostic tool to replace SU or 24 hr urine samples. Our model should be applied to survey data to compute the mean sodium/salt consumption in the population (not in individuals). Empirical evidence suggests that, at the population level, mean estimates based on SU samples and on 24 hr urine samples are similar (Huang et al., 2016; Santos et al., 2020).

### Variables

The predictors we used in the ML model were sex, age (years), weight (kg), height (m), systolic blood pressure (SBP, mmHg), and diastolic blood pressure (DBP, mmHg).

The analyzed surveys collect anthropometric and three blood pressure measurements. These are taken by trained fieldworkers following a standard protocol (World Health Organization, 2021b; Departamento de Epidemiologia. Ministerio de Salud, 2021). We used measured weight and height to compute the BMI (kg/m^2^). We used the mean SBP and mean DBP of the second and third blood pressure measurements (i.e., the first blood pressure measurement was discarded).

The outcome was salt intake as per the INTERSALT equation (Brown et al., 2013). We chose this equation because it has been used by WHO STEPS surveys. There is a specific INTERSALT equation for each sex, and they both include the following variables: age (years), BMI (kg/m^2^), SU sodium (mmol/L), and SU creatinine (mmol/L) (Brown et al., 2013). We used the following sex-specific formulas:

$$Men:\left\{\text{23.51 +}\left[\text{0.45 x }Na_{SU}\right]\text{-}\left[\text{3.09 x }Cr_{SU}\right]\text{+}\left[\text{4.16 x BMI}\right]\text{+}\left[\text{0.22 x age}\right]\right\}$$

$$Women:\left\{\text{3.74 +}\left[\text{0.33 x }Na_{SU}\right]\text{-}\left[\text{2.44 x }Cr_{SU}\right]\text{+}\left[\text{2.42 x BMI}\right]\text{+}\left[\text{2.34 x age}\right]\text{-}\left[\text{0.03 x}{\text{ age}}^{\text{2}}\right]\right\}$$

where the subscript *SU* indicates spot urine, *Na* is sodium, *Cr* is creatinine, and *BMI* is body mass index. Because some STEPS surveys had SU creatinine in mg/dL, these values were multiplied by 0.00884 to obtain SU creatinine in mmol/L. No conversion was needed for sodium in SU samples because all surveys herein included already had urinary sodium in mmol/L. The INTERSALT equation computes 24 hr sodium intake, which is then divided by 17.1 to obtain the salt intake in grams per day (g/d) (Brown et al., 2013). For descriptive purposes, we also computed salt intake based on the Kawasaki et al., 1993, Toft et al., 2014, and Tanaka et al., 2002 equations. Of note, our outcome variable was informed by SU samples and not by 24 hr urine samples (gold standard to assess salt consumption). Results should be interpreted according to this limitation.

### Analysis

#### Data preparation

Our complete-case analysis was restricted to men and nonpregnant women aged between 15 and 69 years because of data availability. We dropped participants with implausible BMI levels (outside the range 10–80 kg/m^2^) or with implausible weight (outside the range 12–300 kg) or height records (outside the range 1.00–2.50 m). Participants with SBP outside the range 70–270 mmHg were discarded, and so were participants with DBP outside the range 30–150 mmHg. We excluded records with SU creatinine <1.8 or > 32.7 mmol/L for males and <1.8 or >28.3 for females (Santos et al., 2019; Paterson et al., 2019). In addition, we excluded participants with estimated salt intake (using the four equations) above or below 3 standard deviations from the equation-specific mean (Appendix 1—figure 1; Jensen et al., 2018). After completing data preparation, observations were randomly assigned from the pooled dataset (100%) into three datasets for the ML analysis: training dataset (50%), test dataset (30%), and validation dataset (20%).

#### Machine learning modeling

Our research aim was a regression problem where we had a known outcome attribute (salt consumption at the subject level). Therefore, we planned a supervised ML regression analysis. Details about the modeling process are available in the ‘Extended methods’ (Appendix 2). In brief, we designed a work pipeline with five steps. First, *data analysis*, where we dropped missing observations, we explored the available data to choose scaling and transformation methods to secure all variables were in the same scale or units, and we also planned transformations for categorical variables (e.g., one-hot encoding). Second, *feature importance analysis*, where we investigated the contribution of each predictor to the regression model through methods like Random Forest (RF) and Recursive Feature Elimination. The aim of this second step was to exclude any predictor that would not contribute to the regression model. Notably, all predictors (see ‘Variables’ section) chosen following expert knowledge were kept in the analysis (i.e., the feature importance analysis did not suggest the exclusion of any predictor). Third, *data processing*, having explored the available data (first step in the work pipeline), we implemented different scaling and transformation methods (e.g., Box-Cox, principal component analysis and polynomial features). Fourth, *data modeling*, where we implemented 10 ML algorithms: (i) linear regression (LiR); (ii) Hubber regressor (HuR); (iii) ridge regressor (RiR); (iv) multilayer perceptron (MLP); (v) support vector regressor (SVR); (vi) *k*-nearest neighbors (KNN); (vii) RF; (viii) gradient boost machine (GBM); (ix) extreme gradient boosting (XBG); and (x) a customized neural network. All these ML algorithms performed similarly, so the decision to choose one was postponed to the fifth (last) step in the work pipeline. Up to this point, we used the training and validations datasets. Five, *forecasting* of the predicted attribute in new data (i.e., data not used for model training); in this step, we used the test dataset to choose the model that yielded predictions closest to the observed salt intake. Results comparing the observed and the predicted salt intake were computed in the test dataset alone. For each country, we ran a paired *t*-test between the observed and predicted salt consumption, where a difference was deemed significant at a p<0.05. We also computed the absolute difference between the observed and predicted salt intake. We chose the HuR algorithm because it showed the mean difference closest to zero in both sexes combined (observed – predicted = 0) (Appendix 2—table 2, Appendix 2—figure 3) . All summary estimates (e.g., mean salt intake) were computed accounting for the complex survey design of the surveys included in the analysis.

#### Application of the developed ML model

Having developed the ML model following the steps above described, we applied the model to 54 WHO STEPS national surveys that did not have urine samples but included the predictors in the ML model (see ‘Variables’ section). In each of these 54 surveys, we computed the mean daily salt intake accounting for the complex survey design. These surveys were preprocessed following the same procedures described in the ‘Data preparation’ section.

#### Role of the funding source

The funder had no role in the study design, analysis, interpretation, or decision to publish. The authors are collectively responsible for the accuracy of the data. The arguments and opinions in this work are those of the authors alone, and do not represent the position of the institutions to which they belong.

## Figures and Tables

**Appendix 1—figure 1.**
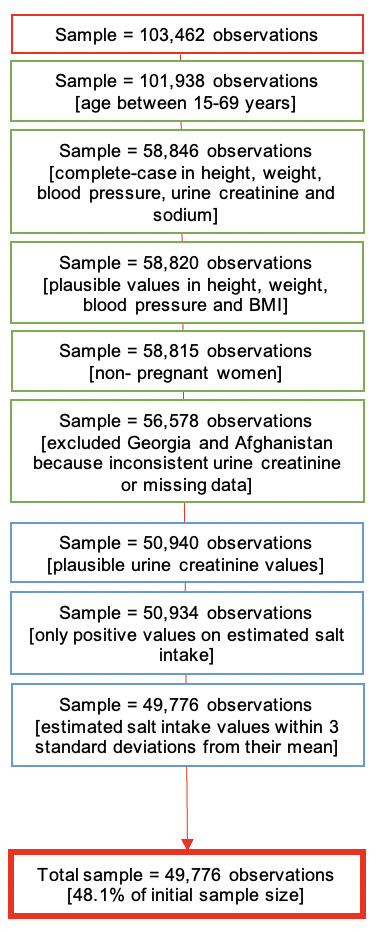
Flowchart of data cleaning and inclusion criteria for model derivation.

**Appendix 2—figure 1.**
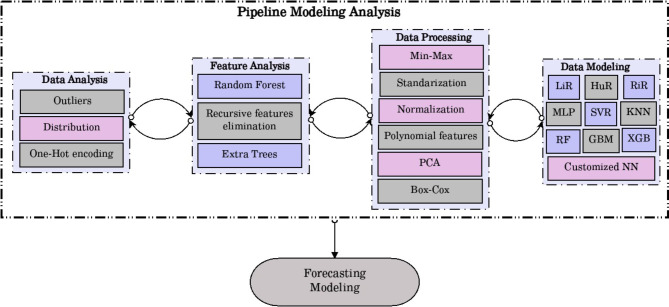
Pipeline for data management and model development. PCA, primary component analysis; LiR, linear regression; HuR, Hubber regressor; RiR, ridge regressor; MLP, multilayer perceptron; SVR, support vector regressor; KNN, *k*-nearest neighbors; RF, random forest; GBM, gradient boost machine; XGB, extreme gradient boosting; NN, neural network.

**Appendix 2—figure 2.**
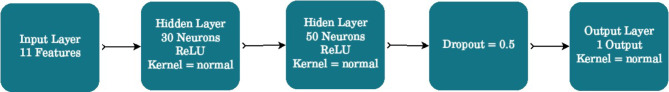
Neural network implementation.

**Appendix 2—figure 3.**
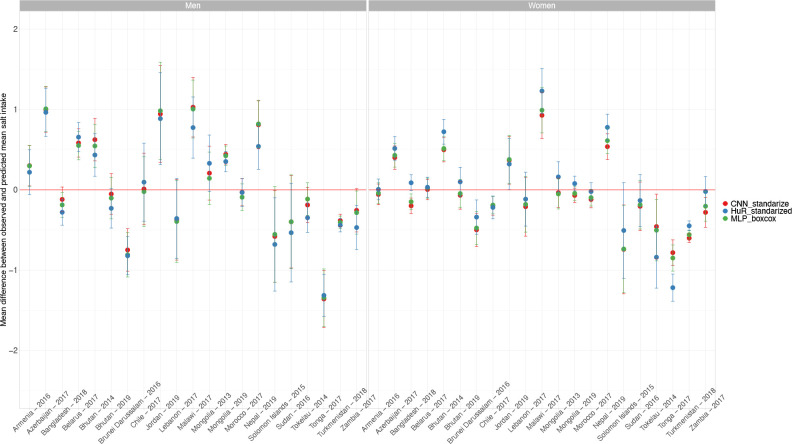
Comparison between mean difference between observed and predicted salt intake across the best algorithms. CNN, customized neural network; HuR: Hubber regressor; MLP, multilayer perceptron.

## Data Availability

This study used nationally-representative survey data that are in the public domain, which was requested through the online repository (https://extranet.who.int/ncdsmicrodata/index.php/home). We provide the analysis code of data preparation and data analysis as supplementary materials to this paper (Source Code File - "Analysis Code | Python and R").

## Additional files

Transparent reporting form

Source code 1. Analysis Code | Python and R.

## Appendix 1

**Appendix 1—table 1.** Weighted distribution of predictors in each survey included in the machine learning model development.

Country	Year	Sample size	Mean age (years)	Age range (years)	Proportion of men (%)	Mean, minimum and maximum values of SBP (mmHg)	Mean, minimum, and maximum values of DBP (mmHg)	Mean, minimum, and maximum values of weight (kg)	Mean, minimum, and maximum values of height (m)	Mean, minimum, and maximum values of urinary sodium (mmol/L)	Mean, minimum, and maximum values of urinary creatinine (mmol/L)
Armenia	2016	1074	40	18–69	49.7	129 (86–238)	85 (49–148)	70.9 (35–139)	1.66 (1.27–1.89)	128.6 (10.6–237.6)	10.1 (1.9–27.3)
Azerbaijan	2017	2359	39	18–69	49.5	126 (82–230)	81 (48–142)	73.1 (36–174)	1.67 (1.15–1.98)	167.7 (2–389)	11.9 (1.8–31.8)
Bangladesh	2018	6200	39	18–69	46.9	121 (72–251)	79 (32–147)	55.9 (28–111)	1.56 (1–2.11)	119.6 (4–422)	8.4 (2.2–32.3)
Belarus	2017	4503	43	18–69	47.1	135 (88–257)	85 (54–147)	77.7 (41–144)	1.7 (1.05–1.99)	149.5 (10.5–371.4)	12.2 (1.8–32.7)
Bhutan	2014	6163	38	18–69	59.2	126 (75–228)	85 (46–142)	61.4 (23–115)	1.6 (1.11–1.96)	142.1 (6–388)	8.1 (1.9–29.7)
Bhutan	2019	6163	34	15–69	56.8	124 (85–224)	82 (44–137)	61.9 (28.5–140)	1.58 (1.07–1.92)	129.9 (4.7–444.9)	10.4 (1.8–32.7)
Brunei Darussalam	2016	1635	35	18–69	51.4	123 (76–218)	78 (46–138)	69 (31.2–138.3)	1.59 (1.32–1.84)	122.6 (19.9–329)	12.6 (1.8–32.6)
Chile	2017	2952	39	15–69	49.8	120 (81–226)	74 (44–130)	75.7 (38.3–146.9)	1.63 (1.34–1.96)	135.8 (10–324)	12.1 (1.8–32.2)
Jordan	2019	1040	37	18–69	50.2	118 (75–200)	78 (50–120)	76.3 (35.5–159.5)	1.66 (1.36–1.95)	165.4 (13–365)	13.6 (1.8–32.5)
Lebanon	2017	998	42	17–69	48.7	129 (80–214)	77 (35–123)	78.3 (40–141)	1.68 (1.2–1.96)	124.4 (4–385)	11.5 (1.9–32)
Malawi	2017	1601	35	18–69	56.4	122 (74–222)	76 (40–142)	58.5 (33.6–119)	1.61 (1.36–1.96)	186.5 (11–399.9)	10.7 (1.9–32.4)
Mongolia	2013	7505	42	15–64	50.3	129 (88–220)	82 (50–134)	71 (30.6–138)	1.62 (1.27–1.92)	134.1 (13.1–515)	10.9 (1.8–31.9)
Mongolia	2019	7505	36	15–69	50.9	120 (76–254)	77 (48–143)	68.4 (29–159)	1.64 (1.34–1.98)	117 (2.1–348.9)	7.5 (1.8–28.3)
Morocco	2017	3435	40	18–69	50.6	128 (83–228)	78 (40–139)	70.9 (35–168)	1.66 (1.34–1.95)	122.3 (26.3–575.2)	10.3 (1.8–31.4)
Nepal	2019	2560	36	15–69	41	124 (81–239)	81 (55–146)	54.6 (26–160)	1.55 (1.21–2.03)	140.9 (3–437)	5.6 (1.8–25.5)
SolomonIslands	2015	172	38	18–69	61.4	121 (88–188)	77 (52–104)	67.9 (38.5–122)	1.61 (1.41–1.8)	99.3 (7–250)	9.7 (1.9–28.4)
Sudan	2016	571	36	18–69	55.9	128 (89–231)	85 (58–132)	72.2 (35.6–174)	1.67 (1.42–1.92)	128.5 (5–459)	14 (1.9–32.4)
Tokelau	2014	181	35	18–63	56	125 (76–184)	79 (53–128)	94.8 (58–158.3)	1.71 (1.16–1.88)	62.4 (20–265)	5 (2–7.7)
Tonga	2017	755	40	18–69	35.7	131 (96–208)	83 (53–148)	98.6 (48.1–181)	1.69 (1.4–1.94)	101.9 (4–327)	15.3 (1.8–32.7)
Turkmenistan	2018	3584	37	18–69	52.7	127 (88–268)	83 (54–149)	72.4 (39–142)	1.68 (1.16–1.98)	109.2 (10–163)	11.1 (4.5–18.3)
Zambia	2017	2488	33	18–69	50.3	125 (73–248)	77 (36–148)	60.9 (33.8–150)	1.62 (1.01–2.07)	137.2 (10–375)	12.2 (1.8–32.4)

**Appendix 1—table 2.** Observed and predicted mean salt intake (g/day) by sex in each survey included in the machine learning model development.

Country	Year	Sex	Mean salt intake	Mean salt intake lower 95% confidence interval	Mean salt intake upper 95% confidence interval	Category
Armenia	2016	Men	9.24	9.04	9.45	ML predicted
Armenia	2016	Men	9.46	9.11	9.81	Observed
Armenia	2016	Women	7.43	7.3	7.57	ML predicted
Armenia	2016	Women	7.44	7.26	7.62	Observed
Azerbaijan	2017	Men	9.43	9.33	9.53	ML predicted
Azerbaijan	2017	Men	10.39	10.06	10.72	Observed
Azerbaijan	2017	Women	7.43	7.31	7.55	ML predicted
Azerbaijan	2017	Women	7.94	7.75	8.14	Observed
Bangladesh	2018	Men	8.87	8.8	8.93	ML predicted
Bangladesh	2018	Men	8.59	8.42	8.75	Observed
Bangladesh	2018	Women	7.18	7.13	7.24	ML predicted
Bangladesh	2018	Women	7.27	7.17	7.37	Observed
Belarus	2017	Men	9.49	9.42	9.56	ML predicted
Belarus	2017	Men	10.14	9.94	10.35	Observed
Belarus	2017	Women	7.53	7.45	7.61	ML predicted
Belarus	2017	Women	7.56	7.41	7.72	Observed
Bhutan	2014	Men	9.14	9.04	9.25	ML predicted
Bhutan	2014	Men	9.58	9.27	9.88	Observed
Bhutan	2014	Women	7.38	7.3	7.46	ML predicted
Bhutan	2014	Women	8.1	7.94	8.27	Observed
Bhutan	2019	Men	9.33	9.25	9.41	ML predicted
Bhutan	2019	Men	9.1	8.85	9.35	Observed
Bhutan	2019	Women	7.43	7.36	7.49	ML predicted
Bhutan	2019	Women	7.53	7.33	7.73	Observed
Brunei Darussalam	2016	Men	9.78	9.57	9.99	ML predicted
Brunei Darussalam	2016	Men	8.95	8.66	9.25	Observed
Brunei Darussalam	2016	Women	7.64	7.5	7.77	ML predicted
Brunei Darussalam	2016	Women	7.3	7.05	7.54	Observed
Chile	2017	Men	9.65	9.56	9.75	ML predicted
Chile	2017	Men	9.75	9.18	10.31	Observed
Chile	2017	Women	7.86	7.8	7.93	ML predicted
Chile	2017	Women	7.64	7.45	7.83	Observed
Jordan	2019	Men	9.31	9.03	9.6	ML predicted
Jordan	2019	Men	10.2	9.52	10.88	Observed
Jordan	2019	Women	7.78	7.53	8.03	ML predicted
Jordan	2019	Women	8.1	7.75	8.45	Observed
Lebanon	2017	Men	9.88	9.62	10.14	ML predicted
Lebanon	2017	Men	9.53	9.06	9.99	Observed
Lebanon	2017	Women	7.63	7.39	7.86	ML predicted
Lebanon	2017	Women	7.51	7.07	7.95	Observed
Malawi	2017	Men	8.76	8.66	8.86	ML predicted
Malawi	2017	Men	9.54	9.16	9.91	Observed
Malawi	2017	Women	7.1	6.97	7.24	ML predicted
Malawi	2017	Women	8.34	8.03	8.64	Observed
Mongolia	2013	Men	9.5	9.32	9.68	ML predicted
Mongolia	2013	Men	9.83	9.34	10.32	Observed
Mongolia	2013	Women	7.63	7.51	7.74	ML predicted
Mongolia	2013	Women	7.79	7.6	7.97	Observed
Mongolia	2019	Men	9.32	9.23	9.42	ML predicted
Mongolia	2019	Men	9.68	9.5	9.85	Observed
Mongolia	2019	Women	7.39	7.32	7.46	ML predicted
Mongolia	2019	Women	7.46	7.34	7.59	Observed
Morocco	2017	Men	9.06	8.97	9.15	ML predicted
Morocco	2017	Men	9.03	8.82	9.24	Observed
Morocco	2017	Women	7.49	7.43	7.56	ML predicted
Morocco	2017	Women	7.47	7.35	7.59	Observed
Nepal	2019	Men	9	8.83	9.18	ML predicted
Nepal	2019	Men	9.55	9.22	9.87	Observed
Nepal	2019	Women	7.07	6.98	7.15	ML predicted
Nepal	2019	Women	7.84	7.65	8.04	Observed
Solomon Islands	2015	Men	9.42	9.25	9.59	ML predicted
Solomon Islands	2015	Men	8.74	8.08	9.4	Observed
Solomon Islands	2015	Women	7.54	7.29	7.79	ML predicted
Solomon Islands	2015	Women	7.03	6.42	7.64	Observed
Sudan	2016	Men	9.07	8.76	9.37	ML predicted
Sudan	2016	Men	8.53	7.73	9.33	Observed
Sudan	2016	Women	7.62	7.27	7.97	ML predicted
Sudan	2016	Women	7.49	7.09	7.88	Observed
Tokelau	2014	Men	10.64	10.34	10.93	ML predicted
Tokelau	2014	Men	10.29	10.18	10.4	Observed
Tokelau	2014	Women	8.96	8.71	9.21	ML predicted
Tokelau	2014	Women	8.12	7.61	8.63	Observed
Tonga	2017	Men	10.5	10.31	10.69	ML predicted
Tonga	2017	Men	9.19	8.89	9.48	Observed
Tonga	2017	Women	8.85	8.65	9.04	ML predicted
Tonga	2017	Women	7.63	7.45	7.81	Observed
Turkmenistan	2018	Men	9.38	9.28	9.48	ML predicted
Turkmenistan	2018	Men	8.94	8.79	9.09	Observed
Turkmenistan	2018	Women	7.2	7.13	7.27	ML predicted
Turkmenistan	2018	Women	6.76	6.68	6.83	Observed
Zambia	2017	Men	8.92	8.84	9	ML predicted
Zambia	2017	Men	8.45	8.15	8.75	Observed
Zambia	2017	Women	7.04	6.96	7.12	ML predicted
Zambia	2017	Women	7.01	6.81	7.22	Observed

ML: machine learning; SBP: systolic blood pressure; DBP: diastolic blood pressure.

**Appendix 1—table 3.** Observed and predicted mean salt intake (g/day) by age, body mass index (BMI) category, and blood pressure status across all surveys included in the machine learning model development dataset.

Attributed	Salt consumption (g/day) observed using surveys included in the derivation model		Salt consumption (g/day) estimated using the surveys included in the derivation model	
	Mean	p-Value for independent *t*-test or ANOVA test	Mean	p-Value for independent *t*-test or ANOVA test
Age <30 years	7.9	<0.001	8.0	< 0.001
Age ≥ 30 years	8.4		8.3	
BMI <18.5 kg/m^2^	7.0	< 0.001	7.0	< 0.001
BMI 18.5–24.9 kg/m^2^	7.8		7.7	
BMI 25.0–29.9 kg/m^2^	8.6		8.4	
BMI ≥ 30 kg/m^2^	9.3		9.3	
Raised blood pressure ( ≥ 140/90 mmHg)	8.7	< 0.001	8.6	< 0.001
No raised blood pressure	8.2		8.1	

These results do not consider the survey sampling design.

**Appendix 1—table 4.** Mean difference (g/day) between observed and predicted salt intake by sex in each survey included in the machine learning (ML) model development.

Country	Year	Sex	Mean difference	Mean difference lower 95% confidence interval	Mean difference upper 95% confidence interval	p-Value
Armenia	2016	Men	0.22	–0.06	0.5	0.0007
Armenia	2016	Women	0.01	–0.12	0.13	0.1953
Azerbaijan	2017	Men	0.96	0.67	1.26	< 0.0001
Azerbaijan	2017	Women	0.52	0.37	0.66	< 0.0001
Bangladesh	2018	Men	–0.28	–0.44	–0.12	< 0.0001
Bangladesh	2018	Women	0.09	–0.01	0.19	0.0004
Belarus	2017	Men	0.66	0.47	0.84	< 0.0001
Belarus	2017	Women	0.03	–0.09	0.16	0.6258
Bhutan	2014	Men	0.43	0.17	0.7	< 0.0001
Bhutan	2014	Women	0.72	0.57	0.88	< 0.0001
Bhutan	2019	Men	–0.23	–0.48	0.02	0.0007
Bhutan	2019	Women	0.1	–0.08	0.28	0.7508
Brunei Darussalam	2016	Men	–0.82	–1.06	–0.58	< 0.0001
Brunei Darussalam	2016	Women	–0.34	–0.55	–0.13	< 0.0001
Chile	2017	Men	0.1	–0.39	0.58	0.0001
Chile	2017	Women	–0.22	–0.36	–0.08	< 0.0001
Jordan	2019	Men	0.89	0.31	1.46	0.0065
Jordan	2019	Women	0.32	0	0.64	0.4142
Lebanon	2017	Men	–0.36	–0.85	0.14	0.2074
Lebanon	2017	Women	–0.12	–0.45	0.22	0.1591
Malawi	2017	Men	0.77	0.39	1.16	< 0.0001
Malawi	2017	Women	1.23	0.95	1.51	< 0.0001
Mongolia	2013	Men	0.33	–0.02	0.68	0.0184
Mongolia	2013	Women	0.16	–0.03	0.35	0.2655
Mongolia	2019	Men	0.35	0.23	0.48	< 0.0001
Mongolia	2019	Women	0.08	–0.01	0.17	0.5155
Morocco	2017	Men	–0.03	–0.21	0.14	0.3083
Morocco	2017	Women	–0.02	–0.13	0.09	0.7259
Nepal	2019	Men	0.54	0.25	0.83	< 0.0001
Nepal	2019	Women	0.78	0.61	0.94	< 0.0001
Solomon Islands	2015	Men	–0.68	–1.26	–0.1	0.0477
Solomon Islands	2015	Women	–0.51	–1.1	0.09	0.0539
Sudan	2016	Men	–0.53	–1.15	0.08	0.2111
Sudan	2016	Women	–0.13	–0.45	0.19	0.0674
Tokelau	2014	Men	–0.35	–0.53	–0.16	0.2248
Tokelau	2014	Women	–0.84	–1.22	–0.45	0.0026
Tonga	2017	Men	–1.31	–1.58	–1.05	< 0.0001
Tonga	2017	Women	–1.22	–1.39	–1.05	< 0.0001
Turkmenistan	2018	Men	–0.44	–0.52	–0.36	< 0.0001
Turkmenistan	2018	Women	–0.45	–0.51	–0.39	< 0.0001
Zambia	2017	Men	–0.47	–0.74	–0.19	< 0.0001
Zambia	2017	Women	–0.02	–0.21	0.17	0.3438

p-Value for paired *t* Student test between observed and predicted.

**Appendix 1—table 5.** Observed mean salt intake (g/day) by equation and sex in each survey included in the machine learning (ML) model development.

Country	Year	Sex	Mean salt intake	Mean salt intake lower 95% confidence interval	Mean salt intake upper 95% confidence interval	Category
Armenia	2016	Men	9.46	9.11	9.81	Observed_intersalt
Armenia	2016	Men	14.58	13.71	15.44	Observed_kawasaki
Armenia	2016	Men	10.21	9.71	10.7	Observed_tanaka
Armenia	2016	Men	12.71	12.19	13.23	Observed_toft
Armenia	2016	Women	7.44	7.26	7.62	Observed_intersalt
Armenia	2016	Women	12.48	11.87	13.09	Observed_kawasaki
Armenia	2016	Women	9.98	9.59	10.36	Observed_tanaka
Armenia	2016	Women	8.41	8.26	8.57	Observed_toft
Azerbaijan	2017	Men	10.39	10.06	10.72	Observed_intersalt
Azerbaijan	2017	Men	14.82	14.21	15.42	Observed_kawasaki
Azerbaijan	2017	Men	10.31	9.98	10.64	Observed_tanaka
Azerbaijan	2017	Men	12.81	12.45	13.18	Observed_toft
Azerbaijan	2017	Women	7.94	7.75	8.14	Observed_intersalt
Azerbaijan	2017	Women	12.65	12.22	13.08	Observed_kawasaki
Azerbaijan	2017	Women	10.14	9.87	10.41	Observed_tanaka
Azerbaijan	2017	Women	8.45	8.33	8.56	Observed_toft
Bangladesh	2018	Men	8.59	8.42	8.75	Observed_intersalt
Bangladesh	2018	Men	12.59	12.25	12.93	Observed_kawasaki
Bangladesh	2018	Men	8.81	8.62	9.01	Observed_tanaka
Bangladesh	2018	Men	11.62	11.4	11.85	Observed_toft
Bangladesh	2018	Women	7.27	7.17	7.37	Observed_intersalt
Bangladesh	2018	Women	12.09	11.78	12.4	Observed_kawasaki
Bangladesh	2018	Women	9	8.82	9.19	Observed_tanaka
Bangladesh	2018	Women	8.33	8.25	8.42	Observed_toft
Belarus	2017	Men	10.14	9.94	10.35	Observed_intersalt
Belarus	2017	Men	14.22	13.85	14.6	Observed_kawasaki
Belarus	2017	Men	10.16	9.95	10.38	Observed_tanaka
Belarus	2017	Men	12.46	12.24	12.69	Observed_toft
Belarus	2017	Women	7.56	7.41	7.72	Observed_intersalt
Belarus	2017	Women	11.43	11.1	11.75	Observed_kawasaki
Belarus	2017	Women	9.59	9.37	9.8	Observed_tanaka
Belarus	2017	Women	8.09	8	8.18	Observed_toft
Bhutan	2014	Men	9.58	9.27	9.88	Observed_intersalt
Bhutan	2014	Men	15.05	14.23	15.87	Observed_kawasaki
Bhutan	2014	Men	10.06	9.64	10.48	Observed_tanaka
Bhutan	2014	Men	13	12.51	13.49	Observed_toft
Bhutan	2014	Women	8.1	7.94	8.27	Observed_intersalt
Bhutan	2014	Women	14.24	13.72	14.76	Observed_kawasaki
Bhutan	2014	Women	10.54	10.22	10.86	Observed_tanaka
Bhutan	2014	Women	8.85	8.72	8.99	Observed_toft
Bhutan	2019	Men	9.1	8.85	9.35	Observed_intersalt
Bhutan	2019	Men	12.81	12.23	13.39	Observed_kawasaki
Bhutan	2019	Men	8.81	8.51	9.11	Observed_tanaka
Bhutan	2019	Men	11.62	11.28	11.97	Observed_toft
Bhutan	2019	Women	7.53	7.33	7.73	Observed_intersalt
Bhutan	2019	Women	11.59	11.22	11.96	Observed_kawasaki
Bhutan	2019	Women	8.9	8.67	9.12	Observed_tanaka
Bhutan	2019	Women	8.18	8.07	8.28	Observed_toft
Brunei Darussalam	2016	Men	8.95	8.66	9.25	Observed_intersalt
Brunei Darussalam	2016	Men	11.51	10.95	12.08	Observed_kawasaki
Brunei Darussalam	2016	Men	8.17	7.89	8.45	Observed_tanaka
Brunei Darussalam	2016	Men	10.79	10.44	11.14	Observed_toft
Brunei Darussalam	2016	Women	7.3	7.05	7.54	Observed_intersalt
Brunei Darussalam	2016	Women	10.52	10.02	11.01	Observed_kawasaki
Brunei Darussalam	2016	Women	8.38	8.08	8.69	Observed_tanaka
Brunei Darussalam	2016	Women	7.88	7.73	8.03	Observed_toft
Chile	2017	Men	9.75	9.18	10.31	Observed_intersalt
Chile	2017	Men	12.86	12.07	13.66	Observed_kawasaki
Chile	2017	Men	9.25	8.84	9.66	Observed_tanaka
Chile	2017	Men	11.66	11.14	12.17	Observed_toft
Chile	2017	Women	7.64	7.45	7.83	Observed_intersalt
Chile	2017	Women	11.11	10.81	11.4	Observed_kawasaki
Chile	2017	Women	9.13	8.93	9.32	Observed_tanaka
Chile	2017	Women	8.06	7.97	8.15	Observed_toft
Jordan	2019	Men	10.2	9.52	10.88	Observed_intersalt
Jordan	2019	Men	13.98	12.73	15.23	Observed_kawasaki
Jordan	2019	Men	9.84	9.17	10.51	Observed_tanaka
Jordan	2019	Men	12.29	11.56	13.02	Observed_toft
Jordan	2019	Women	8.1	7.75	8.45	Observed_intersalt
Jordan	2019	Women	12.1	11.48	12.72	Observed_kawasaki
Jordan	2019	Women	9.74	9.34	10.13	Observed_tanaka
Jordan	2019	Women	8.34	8.17	8.5	Observed_toft
Lebanon	2017	Men	9.53	9.06	9.99	Observed_intersalt
Lebanon	2017	Men	12.72	11.65	13.79	Observed_kawasaki
Lebanon	2017	Men	9.22	8.61	9.84	Observed_tanaka
Lebanon	2017	Men	11.48	10.82	12.14	Observed_toft
Lebanon	2017	Women	7.51	7.07	7.95	Observed_intersalt
Lebanon	2017	Women	11.35	10.45	12.25	Observed_kawasaki
Lebanon	2017	Women	9.37	8.75	10	Observed_tanaka
Lebanon	2017	Women	8.03	7.76	8.3	Observed_toft
Malawi	2017	Men	9.54	9.16	9.91	Observed_intersalt
Malawi	2017	Men	14.02	13.4	14.64	Observed_kawasaki
Malawi	2017	Men	9.43	9.08	9.77	Observed_tanaka
Malawi	2017	Men	12.4	12.04	12.77	Observed_toft
Malawi	2017	Women	8.34	8.03	8.64	Observed_intersalt
Malawi	2017	Women	13.43	12.76	14.11	Observed_kawasaki
Malawi	2017	Women	10.17	9.75	10.58	Observed_tanaka
Malawi	2017	Women	8.64	8.47	8.82	Observed_toft
Mongolia	2013	Men	9.83	9.34	10.32	Observed_intersalt
Mongolia	2013	Men	13.37	12.74	14.01	Observed_kawasaki
Mongolia	2013	Men	9.48	9.13	9.83	Observed_tanaka
Mongolia	2013	Men	12.04	11.64	12.45	Observed_toft
Mongolia	2013	Women	7.79	7.6	7.97	Observed_intersalt
Mongolia	2013	Women	11.92	11.34	12.5	Observed_kawasaki
Mongolia	2013	Women	9.54	9.16	9.92	Observed_tanaka
Mongolia	2013	Women	8.24	8.08	8.4	Observed_toft
Mongolia	2019	Men	9.68	9.5	9.85	Observed_intersalt
Mongolia	2019	Men	14.83	14.49	15.17	Observed_kawasaki
Mongolia	2019	Men	10.14	9.95	10.32	Observed_tanaka
Mongolia	2019	Men	12.84	12.64	13.05	Observed_toft
Mongolia	2019	Women	7.46	7.34	7.59	Observed_intersalt
Mongolia	2019	Women	12.13	11.81	12.44	Observed_kawasaki
Mongolia	2019	Women	9.63	9.43	9.84	Observed_tanaka
Mongolia	2019	Women	8.31	8.23	8.4	Observed_toft
Morocco	2017	Men	9.03	8.82	9.24	Observed_intersalt
Morocco	2017	Men	13.04	12.63	13.44	Observed_kawasaki
Morocco	2017	Men	9.33	9.1	9.56	Observed_tanaka
Morocco	2017	Men	11.75	11.5	12	Observed_toft
Morocco	2017	Women	7.47	7.35	7.59	Observed_intersalt
Morocco	2017	Women	11.72	11.41	12.04	Observed_kawasaki
Morocco	2017	Women	9.48	9.28	9.68	Observed_tanaka
Morocco	2017	Women	8.18	8.09	8.26	Observed_toft
Nepal	2019	Men	9.55	9.22	9.87	Observed_intersalt
Nepal	2019	Men	16.6	15.92	17.27	Observed_kawasaki
Nepal	2019	Men	10.69	10.33	11.04	Observed_tanaka
Nepal	2019	Men	14.04	13.64	14.44	Observed_toft
Nepal	2019	Women	7.84	7.65	8.04	Observed_intersalt
Nepal	2019	Women	15.35	14.82	15.88	Observed_kawasaki
Nepal	2019	Women	10.9	10.57	11.24	Observed_tanaka
Nepal	2019	Women	9.12	8.99	9.25	Observed_toft
Solomon Islands	2015	Men	8.74	8.08	9.4	Observed_intersalt
Solomon Islands	2015	Men	12.99	11.06	14.93	Observed_kawasaki
Solomon Islands	2015	Men	8.87	7.97	9.77	Observed_tanaka
Solomon Islands	2015	Men	11.62	10.43	12.8	Observed_toft
Solomon Islands	2015	Women	7.03	6.42	7.64	Observed_intersalt
Solomon Islands	2015	Women	11.38	8.78	13.98	Observed_kawasaki
Solomon Islands	2015	Women	8.98	7.34	10.61	Observed_tanaka
Solomon Islands	2015	Women	7.95	7.26	8.64	Observed_toft
Sudan	2016	Men	8.53	7.73	9.33	Observed_intersalt
Sudan	2016	Men	11.66	10.66	12.66	Observed_kawasaki
Sudan	2016	Men	8.49	7.91	9.08	Observed_tanaka
Sudan	2016	Men	10.83	10.17	11.5	Observed_toft
Sudan	2016	Women	7.49	7.09	7.88	Observed_intersalt
Sudan	2016	Women	11.3	10.6	12.01	Observed_kawasaki
Sudan	2016	Women	9.31	8.85	9.78	Observed_tanaka
Sudan	2016	Women	8.09	7.89	8.3	Observed_toft
Tokelau	2014	Men	10.29	10.18	10.4	Observed_intersalt
Tokelau	2014	Men	14.33	13.16	15.5	Observed_kawasaki
Tokelau	2014	Men	10.1	9.48	10.72	Observed_tanaka
Tokelau	2014	Men	12.42	11.71	13.14	Observed_toft
Tokelau	2014	Women	8.12	7.61	8.63	Observed_intersalt
Tokelau	2014	Women	11.4	9.85	12.95	Observed_kawasaki
Tokelau	2014	Women	9.71	8.69	10.72	Observed_tanaka
Tokelau	2014	Women	8.15	7.76	8.54	Observed_toft
Tonga	2017	Men	9.19	8.89	9.48	Observed_intersalt
Tonga	2017	Men	10.06	9.17	10.95	Observed_kawasaki
Tonga	2017	Men	7.72	7.22	8.22	Observed_tanaka
Tonga	2017	Men	9.77	9.19	10.35	Observed_toft
Tonga	2017	Women	7.63	7.45	7.81	Observed_intersalt
Tonga	2017	Women	9.37	8.88	9.87	Observed_kawasaki
Tonga	2017	Women	8.41	8.06	8.76	Observed_tanaka
Tonga	2017	Women	7.53	7.37	7.68	Observed_toft
Turkmenistan	2018	Men	8.94	8.79	9.09	Observed_intersalt
Turkmenistan	2018	Men	12.11	11.93	12.3	Observed_kawasaki
Turkmenistan	2018	Men	8.85	8.74	8.96	Observed_tanaka
Turkmenistan	2018	Men	11.2	11.09	11.32	Observed_toft
Turkmenistan	2018	Women	6.76	6.68	6.83	Observed_intersalt
Turkmenistan	2018	Women	10.1	9.93	10.26	Observed_kawasaki
Turkmenistan	2018	Women	8.53	8.41	8.65	Observed_tanaka
Turkmenistan	2018	Women	7.78	7.73	7.83	Observed_toft
Zambia	2017	Men	8.45	8.15	8.75	Observed_intersalt
Zambia	2017	Men	12.7	12.09	13.3	Observed_kawasaki
Zambia	2017	Men	8.8	8.46	9.13	Observed_tanaka
Zambia	2017	Men	11.48	11.11	11.86	Observed_toft
Zambia	2017	Women	7.01	6.81	7.22	Observed_intersalt
Zambia	2017	Women	11.11	10.66	11.56	Observed_kawasaki
Zambia	2017	Women	8.8	8.52	9.09	Observed_tanaka
Zambia	2017	Women	8	7.86	8.13	Observed_toft

**Appendix 1—table 6.** Weighted distribution of predictors in each of the 54 national surveys included in the application of the model herein developed.

Country	Year	Sample size	Mean age (years)	Age range (years)	Proportion of men (%)	Mean, minimum, and maximum values of SBP (mmHg)	Mean, minimum, and maximum values of DBP (mmHg)	Mean, minimum, and maximum values of weight (kg)	Mean, minimum, and maximum values of height (m)
American Samoa	2004	2043	40	25–64	50.3	131 (84–230)	82 (46–134)	100.4 (38.6–219.1)	1.69 (1.36–2.19)
Benin	2015	4841	34	18–69	49.6	126 (74–254)	82 (45–142)	62.3 (30–167)	1.64 (1.21–1.98)
Bahamas	2012	1400	42	24–64	49.9	127 (73–248)	82 (32–140)	84.8 (27.9–184.9)	1.67 (1.15–2.03)
Barbados	2007	282	43	25–69	51.9	122 (86–191)	80 (55–115)	77.5 (40.6–232.1)	1.67 (1.17–1.93)
British Virgin Islands	2009	1067	43	25–64	54.1	130 (81–226)	80 (48–126)	83.2 (39.6–176.9)	1.7 (1.14–2.26)
Botswana	2014	3894	33	15–69	52.1	128 (84–262)	80 (47–148)	63.9 (31.7–171.1)	1.66 (1.02–2)
Cook Islands	2015	879	39	18–64	46.5	128 (92–194)	79 (45–118)	98.6 (49.1–205.1)	1.69 (1.07–1.96)
Comoros	2011	5029	39	25–64	52.6	128 (82–236)	79 (48–144)	64.2 (23.5–166)	1.61 (1–2.15)
Cabo Verde	2007	1723	38	25–64	50.3	133 (86–234)	80 (48–140)	68.3 (35–150)	1.68 (1.23–1.96)
Cayman Islands	2012	1229	42	24–64	50.7	125 (84–208)	76 (46–127)	82.3 (31–196)	1.69 (1–2.1)
Algeria	2017	6536	38	18–69	51.7	127 (77–227)	75 (32–137)	73.3 (25–174)	1.67 (1.02–2.05)
Ecuador	2018	4466	40	18–69	49.4	120 (78–220)	76 (42–130)	69.2 (33.4–198.4)	1.59 (1.24–1.93)
Eritrea	2010	5651	42	25–69	17.2	117 (72–230)	74 (46–130)	51.8 (28.1–99.1)	1.6 (1.16–1.89)
Ethiopia	2015	9270	31	15–69	56.1	120 (71–250)	78 (30–142)	54.4 (20–99.5)	1.63 (1.05–2)
Fiji	2011	2492	42	25–64	51	130 (84–228)	80 (39–143)	78.6 (30.3–198.1)	1.68 (1.03–1.94)
Gambia	2010	3496	38	25–64	50.4	130 (85–252)	80 (44–144)	64.8 (26.5–168.9)	1.64 (1-2)
Grenada	2011	1055	41	25–64	50.7	131 (71–212)	80 (50–128)	77.6 (40.8–158.8)	1.7 (1.32–2.49)
Guyana	2016	2625	37	18–69	52	126 (74–245)	78 (37–149)	69.9 (26.4–198)	1.63 (1.01–2.07)
Iraq	2015	3655	35	18–69	53.6	128 (78–225)	83 (45–150)	76.5 (36.6–187.2)	1.65 (1.01–1.97)
Kenya	2015	4270	34	16–69	50.6	125 (76–262)	81 (46–146)	63.2 (30–171.3)	1.65 (1.01–1.95)
Kyrgyzstan	2013	2539	41	25–64	51.9	133 (82–244)	87 (56–150)	71.7 (36.6–162.4)	1.64 (1.38–1.95)
Cambodia	2010	5223	40	25–64	49.4	116 (70–226)	72 (42–138)	53.7 (21.1–111)	1.57 (1.24–1.85)
Kiribati	2016	1240	40	18–69	42.8	128 (85–220)	85 (49–148)	81.1 (30–219)	1.64 (1.22–1.89)
Kuwait	2014	2871	36	18–69	49.5	120 (70–240)	77 (50–130)	80.5 (37.3–195)	1.65 (1.04–1.96)
Lao People’s Democratic Republic	2013	2464	39	16–65	42.3	119 (72–240)	76 (30–130)	54.2 (27–103.1)	1.54 (1.16–1.97)
Liberia	2011	2242	40	25–64	50.7	129 (88–232)	80 (32–138)	65.4 (32–163)	1.58 (1–2.5)
Libya	2009	3223	37	25–64	51.5	133 (74–238)	79 (44–148)	77 (31.7–186.2)	1.67 (1–1.97)
Sri Lanka	2015	4566	39	18–69	51.5	125 (74–258)	81 (36–150)	58 (26.2–156.9)	1.59 (1.02–1.9)
Lesotho	2012	2162	38	25–64	49.8	126 (78–250)	83 (46–146)	66.2 (21.5–164.6)	1.61 (1.02–1.97)
Republic of Moldova	2013	4077	39	18–69	52.5	133 (83–257)	85 (49–148)	75 (32.5–166)	1.68 (1.2–1.98)
Marshall Islands	2018	2657	39	17–69	48.5	120 (70–220)	75 (40–134)	74.4 (27–226.5)	1.58 (1.01–2.15)
Myanmar	2014	7892	42	25–64	50.4	126 (70–252)	82 (35–144)	57.1 (26.3–173)	1.59 (1–2.18)
Mozambique	2005	723	41	24–64	46	139 (85–220)	82 (46–143)	56.7 (33.4–109.5)	1.6 (1.02–1.89)
Namibia	2005	752	41	25–64	41.3	137 (87–230)	86 (50–132)	63.7 (26.5–134.3)	1.63 (1.12–2)
Niger	2007	2638	37	15–64	54.1	134 (70–260)	82 (40–145)	59.5 (24.3–162.2)	1.67 (1.01–2.1)
Niue	2012	779	40	15–69	50.1	128 (89–223)	76 (44–117)	91.5 (44.7–165.9)	1.69 (1.17–1.96)
Nauru	2016	1037	36	18–69	50	123 (76–223)	80 (46–125)	92.4 (43.4–197.9)	1.63 (1.41–1.86)
Palau	2013	2148	43	25–64	53	138 (87–236)	85 (40–135)	79.4 (32–180.6)	1.62 (1.02–2.03)
French Polynesia	2010	2239	36	18–64	50.7	125 (86–230)	79 (48–150)	86.2 (41–193)	1.7 (1.41–2)
Qatar	2012	2287	35	18–64	50.9	119 (78–203)	79 (46–130)	79.1 (34.4–190.5)	1.64 (1.35–2)
Rwanda	2013	6882	32	15–64	48.8	121 (75–250)	78 (45–140)	57 (23.1–165.8)	1.6 (1–1.91)
Sierra Leone	2009	4473	40	25–64	50.3	131 (72–220)	81 (42–148)	60 (28–185)	1.62 (1–2.34)
Sao Tome and Principe	2008	2272	40	25–64	48.4	135 (78–240)	82 (34–143)	66.1 (30–186.2)	1.64 (1.01–1.98)
Eswatini	2014	3042	31	15–69	47.4	124 (72–252)	80 (42–150)	67.8 (22.2–227.6)	1.63 (1.01–2.02)
Togo	2011	3995	32	15–64	49.3	123 (70–251)	77 (31–142)	61.6 (26–165)	1.64 (1.02–1.99)
Tajikistan	2017	2643	32	18–69	53.8	129 (81–267)	84 (54–150)	66.7 (27.8–148)	1.63 (1.09–2)
Timor-Leste	2014	2480	36	18–69	63.8	130 (72–235)	84 (42–136)	52 (27–165)	1.57 (1.24–1.83)
Tuvalu	2015	1024	39	18–69	54.9	134 (92–246)	84 (48–145)	91.9 (35.8–181.8)	1.68 (1.17–2.06)
United Republic of Tanzania	2012	5381	39	25–64	50.6	129 (80–240)	80 (40–146)	60.6 (29–171.1)	1.63 (1.13–1.97)
Uganda	2014	3673	35	18–69	50.5	125 (83–249)	81 (50–148)	59.4 (30.2–165)	1.62 (1.15–2.03)
Uruguay	2014	2207	38	15–64	47.8	125 (82–232)	79 (44–134)	74.6 (34.3–158)	1.67 (1.36–2.05)
Vietnam	2015	3033	39	18–69	50.4	120 (71–224)	77 (40–128)	54.7 (27.8–106.4)	1.58 (1.01–1.98)
Vanuatu	2011	4420	40	25–64	47.7	130 (77–269)	80 (38–139)	69.4 (28.3–199.8)	1.63 (1.02–2.1)
Samoa	2013	1490	37	18–64	54.1	125 (80–222)	75 (44–132)	90.3 (32.1–160)	1.68 (1.22–1.97)

SBP: systolic blood pressure; DBP: diastolic blood pressure.

**Appendix 1—table 7.** Predicted mean salt intake (g/day) by sex in each of the 54 national surveys included in the application of the model herein developed.

Country	Year	Sex	Mean salt intake	Mean salt intake lower 95% confidence interval	Mean salt intake upper 95% confidence interval
Algeria	2017	Men	9.26	9.22	9.3
Algeria	2017	Women	7.54	7.5	7.58
Algeria	2017	Total	8.43	8.39	8.47
American Samoa	2004	Men	10.9	10.8	10.99
American Samoa	2004	Women	9.03	8.96	9.11
American Samoa	2004	Total	9.97	9.93	10.01
Bahamas	2012	Men	10.09	9.83	10.35
Bahamas	2012	Women	8.11	7.81	8.4
Bahamas	2012	Total	9.1	8.9	9.29
Barbados	2007	Men	9.42	9.25	9.6
Barbados	2007	Women	7.85	7.54	8.17
Barbados	2007	Total	8.67	8.45	8.89
Benin	2015	Men	8.96	8.9	9.03
Benin	2015	Women	7.01	6.89	7.12
Benin	2015	Total	7.98	7.81	8.15
Botswana	2014	Men	8.74	8.68	8.79
Botswana	2014	Women	7.2	7.14	7.26
Botswana	2014	Total	8	7.94	8.06
British Virgin Islands	2009	Men	9.73	9.66	9.81
British Virgin Islands	2009	Women	7.85	7.82	7.88
British Virgin Islands	2009	Total	8.87	8.82	8.92
Cabo Verde	2007	Men	8.98	8.93	9.03
Cabo Verde	2007	Women	7.13	7.03	7.23
Cabo Verde	2007	Total	8.06	7.97	8.16
Cambodia	2010	Men	8.83	8.8	8.86
Cambodia	2010	Women	6.83	6.81	6.86
Cambodia	2010	Total	7.82	7.78	7.86
Cayman Islands	2012	Men	9.73	9.69	9.77
Cayman Islands	2012	Women	7.92	7.61	8.23
Cayman Islands	2012	Total	8.84	8.75	8.92
Comoros	2011	Men	9.06	9.02	9.1
Comoros	2011	Women	7.43	7.38	7.47
Comoros	2011	Total	8.29	8.24	8.33
Cook Islands	2015	Men	10.87	10.73	11.01
Cook Islands	2015	Women	8.74	8.63	8.86
Cook Islands	2015	Total	9.73	9.59	9.88
Ecuador	2018	Men	9.6	9.55	9.65
Ecuador	2018	Women	7.65	7.6	7.69
Ecuador	2018	Total	8.61	8.55	8.68
Eritrea	2010	Men	8.32	8.27	8.37
Eritrea	2010	Women	6.48	6.43	6.52
Eritrea	2010	Total	6.79	6.75	6.84
Eswatini	2014	Men	9.11	9.02	9.2
Eswatini	2014	Women	7.62	7.56	7.68
Eswatini	2014	Total	8.33	8.27	8.39
Ethiopia	2015	Men	8.52	8.49	8.54
Ethiopia	2015	Women	6.62	6.59	6.65
Ethiopia	2015	Total	7.68	7.65	7.72
Fiji	2011	Men	9.53	9.44	9.62
Fiji	2011	Women	7.84	7.76	7.91
Fiji	2011	Total	8.7	8.6	8.8
French Polynesia	2010	Men	10.1	10	10.2
French Polynesia	2010	Women	8	7.9	8.1
French Polynesia	2010	Total	9.06	8.98	9.15
Gambia	2010	Men	9.05	8.94	9.17
Gambia	2010	Women	7.17	7.1	7.25
Gambia	2010	Total	8.12	8.03	8.22
Grenada	2011	Men	9.21	9.12	9.31
Grenada	2011	Women	7.74	7.64	7.84
Grenada	2011	Total	8.49	8.4	8.58
Guyana	2016	Men	9.26	9.16	9.35
Guyana	2016	Women	7.67	7.6	7.74
Guyana	2016	Total	8.5	8.43	8.56
Iraq	2015	Men	9.66	9.58	9.75
Iraq	2015	Women	7.94	7.88	8.01
Iraq	2015	Total	8.87	8.8	8.93
Kenya	2015	Men	8.82	8.73	8.9
Kenya	2015	Women	7.13	7.04	7.21
Kenya	2015	Total	7.98	7.89	8.07
Kiribati	2016	Men	9.92	9.74	10.09
Kiribati	2016	Women	8.27	8.14	8.39
Kiribati	2016	Total	8.97	8.86	9.09
Kuwait	2014	Men	10.06	9.99	10.12
Kuwait	2014	Women	7.95	7.91	8
Kuwait	2014	Total	8.99	8.94	9.05
Kyrgyzstan	2013	Men	9.45	9.34	9.55
Kyrgyzstan	2013	Women	7.62	7.56	7.67
Kyrgyzstan	2013	Total	8.57	8.5	8.63
Lao People’s Democratic Republic	2013	Men	9.03	8.98	9.08
Lao People’s Democratic Republic	2013	Women	7.07	7.02	7.12
Lao People’s Democratic Republic	2013	Total	7.9	7.83	7.97
Lesotho	2012	Men	9.08	8.99	9.17
Lesotho	2012	Women	7.7	7.6	7.79
Lesotho	2012	Total	8.38	8.31	8.46
Liberia	2011	Men	9.43	9.32	9.55
Liberia	2011	Women	7.58	7.48	7.69
Liberia	2011	Total	8.52	8.41	8.63
Libya	2009	Men	9.51	9.44	9.59
Libya	2009	Women	7.81	7.73	7.89
Libya	2009	Total	8.69	8.63	8.75
Marshall Islands	2018	Men	9.92	9.86	9.99
Marshall Islands	2018	Women	8.16	8.1	8.21
Marshall Islands	2018	Total	9.01	8.96	9.07
Mozambique	2005	Men	8.72	8.62	8.83
Mozambique	2005	Women	6.92	6.84	7
Mozambique	2005	Total	7.75	7.63	7.87
Myanmar	2014	Men	8.81	8.74	8.88
Myanmar	2014	Women	7.07	6.97	7.17
Myanmar	2014	Total	7.95	7.88	8.02
Namibia	2005	Men	8.74	8.59	8.89
Namibia	2005	Women	7.24	6.93	7.56
Namibia	2005	Total	7.86	7.63	8.09
Nauru	2016	Men	10.98	10.87	11.1
Nauru	2016	Women	8.79	8.63	8.94
Nauru	2016	Total	9.89	9.74	10.03
Niger	2007	Men	8.56	8.52	8.6
Niger	2007	Women	6.67	6.63	6.71
Niger	2007	Total	7.69	7.65	7.74
Niue	2012	Men	10.39	10.28	10.51
Niue	2012	Women	8.39	8.27	8.51
Niue	2012	Total	9.4	9.29	9.5
Palau	2013	Men	10.18	10.07	10.28
Palau	2013	Women	7.99	7.9	8.08
Palau	2013	Total	9.15	9.05	9.25
Qatar	2012	Men	10.02	9.93	10.11
Qatar	2012	Women	7.94	7.85	8.04
Qatar	2012	Total	9	8.9	9.09
Republic of Moldova	2013	Men	9.51	9.45	9.57
Republic of Moldova	2013	Women	7.46	7.41	7.52
Republic of Moldova	2013	Total	8.54	8.48	8.6
Rwanda	2013	Men	8.87	8.85	8.9
Rwanda	2013	Women	7.02	6.99	7.05
Rwanda	2013	Total	7.92	7.89	7.96
Samoa	2013	Men	10.23	10.09	10.37
Samoa	2013	Women	8.61	8.51	8.71
Samoa	2013	Total	9.49	9.41	9.57
Sao Tome and Principe	2008	Men	9.05	8.97	9.12
Sao Tome and Principe	2008	Women	7.21	7.1	7.32
Sao Tome and Principe	2008	Total	8.1	7.99	8.2
Sierra Leone	2009	Men	8.85	8.76	8.94
Sierra Leone	2009	Women	7	6.9	7.11
Sierra Leone	2009	Total	7.93	7.82	8.04
Sri Lanka	2015	Men	8.91	8.86	8.95
Sri Lanka	2015	Women	7.07	7.03	7.1
Sri Lanka	2015	Total	8.01	7.97	8.06
Tajikistan	2017	Men	9.41	9.34	9.49
Tajikistan	2017	Women	7.35	7.3	7.41
Tajikistan	2017	Total	8.46	8.38	8.55
Timor-Leste	2014	Men	8.91	8.79	9.02
Timor-Leste	2014	Women	6.8	6.75	6.86
Timor-Leste	2014	Total	8.15	7.86	8.43
Togo	2011	Men	8.82	8.79	8.86
Togo	2011	Women	7.01	6.96	7.06
Togo	2011	Total	7.9	7.85	7.96
Tuvalu	2015	Men	10.37	10.24	10.5
Tuvalu	2015	Women	8.72	8.62	8.83
Tuvalu	2015	Total	9.63	9.53	9.73
Uganda	2014	Men	8.8	8.76	8.84
Uganda	2014	Women	7.02	6.96	7.07
Uganda	2014	Total	7.92	7.86	7.98
United Republic of Tanzania	2012	Men	8.71	8.63	8.79
United Republic of Tanzania	2012	Women	7.13	7.05	7.21
United Republic of Tanzania	2012	Total	7.93	7.88	7.98
Uruguay	2014	Men	9.55	9.48	9.63
Uruguay	2014	Women	7.47	7.41	7.52
Uruguay	2014	Total	8.46	8.39	8.53
Vanuatu	2011	Men	9.38	9.33	9.43
Vanuatu	2011	Women	7.45	7.4	7.5
Vanuatu	2011	Total	8.37	8.31	8.43
Vietnam	2015	Men	8.91	8.86	8.95
Vietnam	2015	Women	6.84	6.81	6.88
Vietnam	2015	Total	7.88	7.83	7.94

**Appendix 1—table 8.** Comparison between mean salt intake (g/day) predictions and global estimates across national surveys included in the application of our machine learning model.

Country	Year (machine learning predictions)	Machine learning predicted mean salt intake and 95% confidence interval	Year (global estimates)	Estimated mean salt intake and 95% confidence interval	Ratio between machine learning predicted and global estimates
Algeria	2017	8.4 (8.4–8.5)	2010	10.7 (9–12.5)	0.8
Bahamas	2012	9.1 (8.9–9.3)	2010	7.5 (6.2–8.8)	1.2
Barbados	2007	8.7 (8.4–8.9)	2010	8.6 (7.8–9.4)	1
Benin	2015	8 (7.8–8.2)	2010	7.1 (6.2–8.1)	1.1
Botswana	2014	8 (7.9–8.1)	2010	6.3 (5.4–7.4)	1.3
Cabo Verde	2007	8.1 (8–8.2)	2010	8.1 (6.8–9.7)	1
Cambodia	2010	7.8 (7.8–7.9)	2010	11 (9.3–12.9)	0.7
Comoros	2011	8.3 (8.2–8.3)	2010	4.2 (3.5–5)	2
Ecuador	2018	8.6 (8.6–8.7)	2010	7.6 (6.4–8.9)	1.1
Eritrea	2010	6.8 (6.8–6.8)	2010	5.9 (5–7)	1.2
Ethiopia	2015	7.7 (7.7–7.7)	2010	5.7 (4.9–6.7)	1.4
Fiji	2011	8.7 (8.6–8.8)	2010	7.2 (6–8.5)	1.2
Gambia	2010	8.1 (8–8.2)	2010	7.7 (6.5–8.9)	1.1
Grenada	2011	8.5 (8.4–8.6)	2010	6.5 (5.5–7.7)	1.3
Guyana	2016	8.5 (8.4–8.6)	2010	6.1 (5.1–7.3)	1.4
Iraq	2015	8.9 (8.8–8.9)	2010	9.4 (8–11.2)	0.9
Kenya	2015	8 (7.9–8.1)	2010	3.7 (3.4–4)	2.2
Kiribati	2016	9 (8.9–9.1)	2010	5.6 (4.6–6.7)	1.6
Kuwait	2014	9 (8.9–9.1)	2010	9.7 (8.7–10.8)	0.9
Kyrgyzstan	2013	8.6 (8.5–8.6)	2010	13.4 (11.4–15.8)	0.6
Lao People’s Democratic Republic	2013	7.9 (7.8–8)	2010	11.1 (9.4–13.2)	0.7
Lesotho	2012	8.4 (8.3–8.5)	2010	6.6 (5.5–7.8)	1.3
Liberia	2011	8.5 (8.4–8.6)	2010	6.7 (5.6–7.9)	1.3
Libya	2009	8.7 (8.6–8.8)	2010	10.6 (8.9–12.5)	0.8
Marshall Islands	2018	9 (9–9.1)	2010	6.4 (5.4–7.5)	1.4
Mozambique	2005	7.8 (7.6–7.9)	2010	5.6 (4.7–6.6)	1.4
Myanmar	2014	8 (7.9–8)	2010	11.2 (9.4–13.2)	0.7
Namibia	2005	7.9 (7.6–8.1)	2010	6.6 (5.6–7.7)	1.2
Niger	2007	7.7 (7.7–7.7)	2010	7.3 (6.2–8.6)	1.1
Qatar	2012	9 (8.9–9.1)	2010	10.5 (8.3–12.9)	0.9
Republic of Moldova	2013	8.5 (8.5–8.6)	2010	9.9 (8.3–11.6)	0.9
Rwanda	2013	7.9 (7.9–8)	2010	4 (3.3–4.9)	2
Samoa	2013	9.5 (9.4–9.6)	2010	5.2 (4.6–5.8)	1.8
Sao Tome and Principe	2008	8.1 (8–8.2)	2010	5.9 (4.9–6.9)	1.4
Sierra Leone	2009	7.9 (7.8–8)	2010	6.3 (5.3–7.3)	1.3
Sri Lanka	2015	8 (8–8.1)	2010	9.7 (8.2–11.3)	0.8
Tajikistan	2017	8.5 (8.4–8.6)	2010	13.5 (11.6–15.7)	0.6
Timor-Leste	2014	8.2 (7.9–8.4)	2010	11.2 (9.3–13.3)	0.7
Uganda	2014	7.9 (7.9–8)	2010	5.3 (4.4–6.3)	1.5
United Republic of Tanzania	2012	7.9 (7.9–8)	2010	6.9 (6.1–7.7)	1.1
Uruguay	2014	8.5 (8.4–8.5)	2010	6.8 (5.8–8)	1.2
Vanuatu	2011	8.4 (8.3–8.4)	2010	5.6 (4.8–6.6)	1.5
Vietnam	2015	7.9 (7.8–7.9)	2010	11.5 (9.5–13.7)	0.7

There are 43 countries in this table; that is, countries included in our analysis that were not available in the previous global work were not included in this table (Powles et al., 2013).

**Appendix 1—table 9.** Countries included in the analysis by income group according to the World Bank classification.

Analysis	World region	Country	Year	Income group
Model application	Africa	Algeria	2017	Upper-middle
Model application	Western Pacific	American Samoa	2004	Upper-middle
Model application	Americas	Bahamas	2012	High
Model application	Americas	Barbados	2007	High
Model application	Africa	Benin	2015	Lower
Model application	Africa	Botswana	2014	Upper-middle
Model application	Americas	British Virgin Islands	2009	No data
Model application	Africa	Cabo Verde	2007	Lower-middle
Model application	Western Pacific	Cambodia	2010	Lower
Model application	Americas	Cayman Islands	2012	High
Model application	Africa	Comoros	2011	Lower
Model application	Western Pacific	Cook Islands	2015	No data
Model application	Americas	Ecuador	2018	Upper-middle
Model application	Africa	Eritrea	2010	Lower
Model application	Africa	Eswatini	2014	Lower-middle
Model application	Africa	Ethiopia	2015	Lower
Model application	Western Pacific	Fiji	2011	Lower-middle
Model application	Western Pacific	French Polynesia	2010	High
Model application	Africa	Gambia	2010	Lower
Model application	Americas	Grenada	2011	Upper-middle
Model application	Americas	Guyana	2016	Upper-middle
Model application	Eastern Mediterranean	Iraq	2015	Upper-middle
Model application	Africa	Kenya	2015	Lower-middle
Model application	Western Pacific	Kiribati	2016	Lower-middle
Model application	Eastern Mediterranean	Kuwait	2014	High
Model application	Eastern Mediterranean	Kyrgyzstan	2013	Lower-middle
Model application	Western Pacific	Lao People’s Democratic Republic	2013	Lower-middle
Model application	Africa	Lesotho	2012	Lower-middle
Model application	Africa	Liberia	2011	Lower
Model application	Eastern Mediterranean	Libya	2009	Upper-middle
Model application	Western Pacific	Marshall Islands	2018	Upper-middle
Model application	Africa	Mozambique	2005	Lower
Model application	Southeast Asia	Myanmar	2014	Lower-middle
Model application	Africa	Namibia	2005	Lower-middle
Model application	Western Pacific	Nauru	2016	Upper-middle
Model application	Africa	Niger	2007	Lower
Model application	Western Pacific	Niue	2012	No data
Model application	Western Pacific	Palau	2013	Upper-middle
Model application	Eastern Mediterranean	Qatar	2012	High
Model application	Europe	Republic of Moldova	2013	Lower-middle
Model application	Africa	Rwanda	2013	Lower
Model application	Western Pacific	Samoa	2013	Lower-middle
Model application	Africa	Sao Tome and Principe	2008	Lower-middle
Model application	Africa	Sierra Leone	2009	Lower
Model application	Southeast Asia	Sri Lanka	2015	Lower-middle
Model application	Europe	Tajikistan	2017	Lower
Model application	Southeast Asia	Timor-Leste	2014	Lower-middle
Model application	Africa	Togo	2011	Lower
Model application	Western Pacific	Tuvalu	2015	Upper-middle
Model application	Africa	Uganda	2014	Lower
Model application	Africa	United Republic of Tanzania	2012	Lower
Model application	Americas	Uruguay	2014	High
Model application	Western Pacific	Vanuatu	2011	Lower-middle
Model application	Western Pacific	Vietnam	2015	Lower-middle
Model derivation	Europe	Armenia	2016	Lower-middle
Model derivation	Europe	Azerbaijan	2017	Upper-middle
Model derivation	Southeast Asia	Bangladesh	2018	Lower-middle
Model derivation	Europe	Belarus	2017	Upper-middle
Model derivation	Southeast Asia	Bhutan	2014	Lower-middle
Model derivation	Southeast Asia	Bhutan	2019	Lower-middle
Model derivation	Western Pacific	Brunei Darussalam	2016	High
Model derivation	Americas	Chile	2017	High
Model derivation	Eastern Mediterranean	Jordan	2019	Upper-middle
Model derivation	Eastern Mediterranean	Lebanon	2017	Upper-middle
Model derivation	Africa	Malawi	2017	Lower
Model derivation	Western Pacific	Mongolia	2013	Lower-middle
Model derivation	Western Pacific	Mongolia	2019	Lower-middle
Model derivation	Eastern Mediterranean	Morocco	2017	Lower-middle
Model derivation	Southeast Asia	Nepal	2019	Lower-middle
Model derivation	Western Pacific	Solomon Islands	2015	Lower-middle
Model derivation	Eastern Mediterranean	Sudan	2016	Lower-middle
Model derivation	Western Pacific	Tokelau	2014	No data
Model derivation	Western Pacific	Tonga	2017	Upper-middle
Model derivation	Europe	Turkmenistan	2018	Upper-middle
Model derivation	Africa	Zambia	2017	Lower-middle

Source: World Bank (https://datahelpdesk.worldbank.org/knowledgebase/articles/906519-world-bank-country-and-lending-groups).

## Appendix 2

### Expanded methods

#### Characteristics of the surveys included in the analysis

We analyzed WHO STEPS surveys and one national health survey (Chile) (World Health Organization, 2021b; Wang et al., 2020). These surveys included a random sample of the general population and can deliver nationally representative estimates. These are household surveys that stratify by the first administrative level in the country (e.g., region); within this level, further stratification may occur by, for example, urban/rural location. Then, a random sample of census tracts, villages, neighborhoods, or other similar division is selected. In each of these primary sampling units, households are randomly sampled for the interview.

All surveys followed standard procedures (World Health Organization, 2021b; Wang et al., 2020). Briefly, participants were given a small container along with instructions for the urine collection; the next day, participants brought the urine sample to a designated place. Then, urine samples were analyzed at a laboratory by a trained technician.

#### Overview

We worked with a structured dataset that mostly had numeric attributes (variables). Given our study problem, we opted for a supervised learning model because there was a target attribute (i.e., salt consumption at the subject level); specifically, we conducted a supervised regression because the target attribute was a numeric variable. For the machine learning analyses, we used Python and the Scikit-Learn library.

First, we developed a pipeline for data management and model development. This way, we followed a consistent and transparent methodology to secure an optimal model for the training set and that would adequately generalize to other (unseen) datasets. Appendix 2—figure 1 depicts the pipeline we developed: (i) we studied the available data and where needed, we did a one-hot encoding; (ii) we did feature importance analysis; (iii) we chose and tried different scaling and transformation methods, so that all variables would be in the same scale or units; (iv) we tried a set of machine learning models, including a customized neural network; and (v) we forecasted (predicted) the attribute of interest (salt consumption at the subject level) in an unseen dataset (i.e., not used for model training). Notably, we went backward and forward (see arrows in the figure) between the four first stages until we reached the best combinations and results for each model. In the following sections, we will describe each of these five stages.

#### Data analysis

This was an exploratory analysis to understand the dataset and its characteristics. We worked with a complete-case dataset; in other words, we excluded missing observations in the variables considered in the analysis. Consequently, we did not do any data imputation analysis.

We explored the distribution of all numerical variables, which were in different units and scales; this exploratory analysis informed the choices of data processing methods (e.g., Box-Cox) implemented in the third stage.

#### Feature importance analysis

Even though we followed expert knowledge to select a reduced, though relevant number of predictors to be included in the regression model, we conducted feature importance analyses to understand the role each predictor would play in the model. This process aimed to eliminate variables that would not carry substantial information for the model. We used random forest, recursive feature elimination, and extra trees. Consistently, these three methods suggested that all the chosen predictors would contribute to a better model.

#### Data processing

As described in the data analysis section (first stage), numeric variables were in different units and scales; therefore, these variables needed to be scaled or transformed. This scaling would also help to find a better prediction model. It is common knowledge that machine learning models would perform differently (and better) depending on data transformation methods. We did (i) min-max whereby numeric variables were scaled to a range between 0 and 1; (ii) standardization; (iii) normalization: (iv) polynomial features of degree 2 (quadratic polynomial); (v) principal component analysis with three components and explained variance of ≥0.95; and (vi) Box-Cox.

#### Data modeling

There are several machine learning algorithms for a supervised regression model. Those that we used, and that are depicted in Appendix 1—figure 1, yielded much better results and were studied in detail. That is, at the beginning of our work we explored other algorithms, though these did not perform well and were not considered thereafter. The algorithms we considered were (i) linear regression (LiR); (ii) Hubber regressor (HuR); (iii) ridge regressor (RiR); (iv) multilayer perceptron (MLP); (v) support vector regressor (SVR); (vi) *k*-nearest neighbors (KNN); (vii) random forest (RF); (viii) gradient boost machine (GBM); and (ix) extreme gradient boosting (XBG).

In addition to these nine machine learning algorithms, we also implemented a neural network (see Appendix 2—figure 2). This neural network was optimized empirically. We used a batch size = 256; epochs = 300; and optimizer = ‘adam.’ The neural network was implemented in Python using the Keras library.

For each model and processing method (see ‘Data processing’ section), we studied the R^2^, mean absolute error (MAE), and root mean square error (RMSE). As shown in Appendix 1—table 1, all algorithms showed a similar performance. Because all the algorithms had an equivalent performance, the chosen one needed to be defined at the forecasting stage; that is, the one that would generalize better to new (unseen) data.

#### Forecasting modeling

This stage implies studying the predicted results in new (unseen) data (i.e., data not used for model training). For this stage, we used the validation and test datasets. We chose the model that yielded predictions closest to the observed results. In this line, we compared the mean difference between the observed and predicted mean salt intake results (i.e., observed – predicted) across all prediction algorithms.

We observed there was no unique algorithm that had the mean difference closest to zero in men and women at the same time (Appendix 1—table 2). The HuR algorithm had the mean difference closest to zero in both sexes combined (mean difference = –0.0019), the RiR algorithm performed the best in men (mean difference = 0.0063), and in women the HuR algorithm showed the best results (mean difference = 0.0082).

To support our decision process, we plotted the mean differences in men and women for each survey (Appendix 2—figure 3); this figure only included the predictions based on the top three algorithms (HuR, MLP, and customized NN). We counted how many times (i.e., number of surveys) each algorithm had the mean difference closest to zero.

Because the HuR algorithm had the mean difference closest to zero in both sexes combined and it was among the top five algorithms in men and women (Appendix 1—table 2), we decided to choose the HuR algorithm. Additionally, predictions based on the HuR algorithm were the closest to zero across surveys (Appendix 2—figure 3). These analyses were performed in R (version 4.0.3).

#### Algorithm application

To make the predictions in the new 54 datasets without information about urine samples, we used the HuR model (i.e., ML algorithm and predictors) developed following the methods above described (see ‘Forecasting modeling’ section). We re-trained the model with the full dataset used for model development and validation (i.e., train, validated, and test dataset pooled), and then predicted the outcome (i.e., mean salt intake) in the 54 new datasets.

**Appendix 2—table 1.** Performance of each algorithm and processing method.

Algorithm	Processing	R^2^	MAE	RMSE
LiR	Polynomial (g = 2)	0.447	1.1138	1.4451
HuR	Standardized	0.447	1.1132	1.4442
RiR	Polynomial (g = 2)	0.446	1.1147	1.4459
MLP	Min-max	0.451	1.1101	1.4395
SVR	Min-max	0.446	1.0988	1.4459
KNN	Standardized	0.421	1.1426	1.4779
RF	Polynomial (g = 2)	0.417	1.1474	1.4835
GBM	Min-max	0.447	1.1147	1.4447
XGB	Min-max	0.431	1.1293	1.4646
Customized NN	Box-Cox	0.461	1.0953	1.4156

MAE: mean absolute error; RMSE: root mean square error; LiR: linear regression; HuR: Hubber regressor; RiR: ridge regressor; MLP: multilayer perceptron; SVR: support vector regressor; KNN: *k-*nearest neighbors; GBM: gradient boost machine; XGB: extreme gradient boosting; NN: neural network; RF: random forest.

**Appendix 2—table 2.** Mean difference between observed and predicted salt intake by sex across all machine learning algorithms.

Machine learning algorithm	Mean difference between observed and predicted mean salt intake	Sex
CNN_boxcox	–0.0109	Both sexes
CNN_standardize	–0.0075	Both sexes
GBR_boxcox	0.1373	Both sexes
GBR_minmax	0.1198	Both sexes
GBR_orig	–0.0252	Both sexes
GBR_standardized	0.1231	Both sexes
HuR_boxcox	0.0389	Both sexes
HuR_standardized	–0.0019	Both sexes
KNN_boxcox	0.0144	Both sexes
KNN_standardized	–0.0172	Both sexes
LiR_poly	–0.0292	Both sexes
MLP_boxcox	–0.0069	Both sexes
MLP_minmax	–0.019	Both sexes
MLP_standardized	–0.0174	Both sexes
RF_poly	–0.0479	Both sexes
RiR_poly	–0.0304	Both sexes
SVR_minmax	0.1137	Both sexes
XGB_boxcox	0.0389	Both sexes
XGB_orig	–0.0312	Both sexes
XGB_standardized	–0.0329	Both sexes
CNN_boxcox	0.088	Men
CNN_standardize	0.0699	Men
GBR_boxcox	0.1591	Men
GBR_minmax	0.1381	Men
GBR_orig	0.0197	Men
GBR_standardized	0.1444	Men
HuR_boxcox	0.0265	Men
HuR_standardized	–0.0119	Men
KNN_boxcox	0.0612	Men
KNN_standardized	0.0179	Men
LiR_poly	0.0069	Men
MLP_boxcox	0.0512	Men
MLP_minmax	–0.0104	Men
MLP_standardized	–0.0249	Men
RF_poly	–0.0129	Men
RiR_poly	0.0063	Men
SVR_minmax	0.1265	Men
XGB_boxcox	0.0265	Men
XGB_orig	0.0147	Men
XGB_standardized	0.0069	Men
CNN_boxcox	–0.1097	Women
CNN_standardized	–0.085	Women
GBR_boxcox	0.1155	Women
GBR_minmax	0.1015	Women
GBR_orig	–0.07	Women
GBR_standardized	0.1018	Women
HuR_boxcox	0.0514	Women
HuR_standardized	0.0082	Women
KNN_boxcox	–0.0324	Women
KNN_standardized	–0.0524	Women
LiR_poly	–0.0653	Women
MLP_boxcox	–0.0649	Women
MLP_minmax	–0.0276	Women
MLP_standardized	–0.0098	Women
RF_poly	–0.0828	Women
RiR_poly	–0.0671	Women
SVR_minmax	0.101	Women
XGB_boxcox	0.0514	Women
XGB_orig	–0.0771	Women
XGB_standardized	–0.0727	Women
